# Association of *ABCB1* and *SLC22A16* Gene Polymorphisms with Incidence of Doxorubicin-Induced Febrile Neutropenia: A Survey of Iranian Breast Cancer Patients

**DOI:** 10.1371/journal.pone.0168519

**Published:** 2016-12-30

**Authors:** Abolfazl Faraji, Hamid Reza Dehghan Manshadi, Maryam Mobaraki, Mahkameh Zare, Massoud Houshmand

**Affiliations:** 1 Department of Medical Genetics, National Institute of Genetic Engineering and Biotechnology, Tehran, Iran; 2 Department of Radiation Oncology, 7-Tir Hospital, Iran University of Medical Sciences, Tehran, Iran; University of South Alabama Mitchell Cancer Institute, UNITED STATES

## Abstract

Breast cancer is the most common cancer in women worldwide. Doxorubicin-based chemotherapy is used to treat breast cancer patients; however, neutropenia is a common hematologic side effect and can be life-threatening. The *ABCB1* and *SLC22A16* genes encode proteins that are essential for doxorubicin transport. In this study, we explored the effect of 2 common polymorphisms in *ABCB1 (rs10276036 C/T)* and *SLC22A16 (rs12210538 A/G)* on the development of grade 3/4 febrile neutropenia in Iranian breast cancer patients. Our results showed no significant association between these polymorphisms and grade 3/4 febrile neutropenia; however, allele *C* of *ABCB1 (rs10276036 C/T)* (p = 0.315, OR = 1.500, 95% CI = 0.679–3.312) and allele *A* of *SLC22A16 (rs12210538 A/G)* (p = 0.110, OR = 2.984, 95% CI = 0.743–11.988) tended to have a greater association with grade 3/4 febrile neutropenia, whereas allele *T* of *ABCB1 (rs10276036)* (p = 0.130, OR = 0.515, 95% CI = 0.217–1.223) and allele *G* of *SLC22A16 (rs12210538)* (p = 0.548, OR = 0.786, 95% CI = 0.358–1.726) tended to protect against this condition. In addition to breast cancer, a statistically significant association was also observed between the development of grade 3/4 febrile neutropenia and other clinical manifestations such as stage IIIC cancer (p = 0.037) and other diseases (p = 0.026). Our results indicate that evaluation of the risk of grade 3/4 neutropenia development and consideration of molecular and clinical findings may be of value when screening for high-risk breast cancer patients.

## Introduction

Breast cancer is the most incident cancer type among women. Doxorubicin (DOX)-based treatments are appropriate for many adult and pediatric solid tumors (including breast cancer), leukemias and lymphomas [1]. However, optimized administration of DOX is hampered owing to some toxicities, such as hematopoietic suppression, nausea, vomiting, and cardiotoxicity [2].

**Fig 1:**
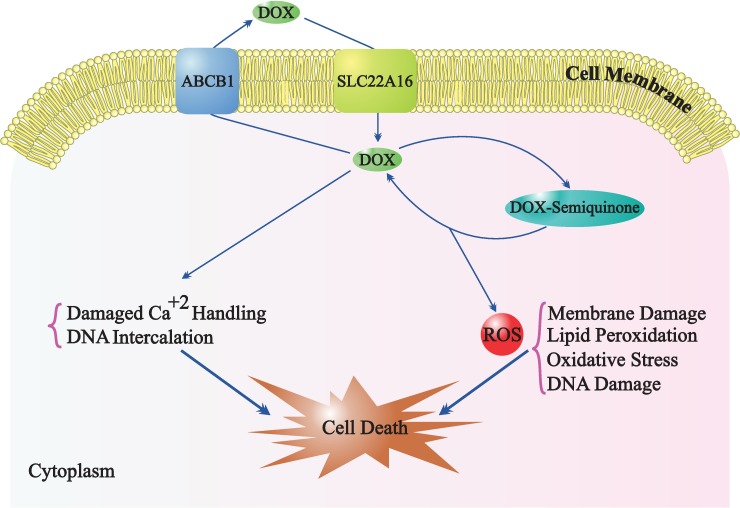
Transport and mechanism of action of doxorubicin.

DOX is a secondary metabolite produced by *Streptomyces peucetius var*. *caesius* and belongs to the family of anthracyclines [3]. DOX functions through dual mechanisms i) intercalation into DNA and disruption of the DNA repair mechanism that is mediated by topoisomerase II, and ii) releasing of free radicals resulting in the damaging of cell membranes, DNA, and proteins [4]. DOX is oxidized to a semiquinone, an unstable metabolite, which is reconverted to DOX through a pathway that releases reactive oxygen species; this can cause lipid peroxidation, membrane damage, DNA damage, oxidative stress, and cell death via induction of apoptotic pathways (Fig 1) [5].

Administration of DOX causes myelosuppression and leads to anemia, thrombocytopenia, and leukopenia. Neutropenia causes immune system suppression, exposing the patient to severe life-threating infections; it is the most serious hematologic toxicity associated with cancer chemotherapy, and its degree and duration determine the risk of infection. Prophylaxis with granulocyte-colony stimulating factor reduces the intensity and duration of chemotherapy-induced neutropenia and attenuates febrile neutropenia risk, and therefore plays a significant role in supporting myelosuppressive chemotherapy [6].

A number of factors have been shown to impact the response to DOX-based chemotherapy, including tumor stage, grade, the number of involved lymph nodes, and expression levels of estrogen receptor (ER), progesterone receptor (PR), and human epidermal growth factor receptor 2 (Her2) [7]. However, inter-patient variability has created a major obstacle for the clinical use of anticancer drugs [8]. Studies showed that these variations may be caused by differences in metabolizing enzymes and transporters associated with DOX. DOX is transported by the protein encoded by the *ABCB1* gene [9–11] as well as the solute transporter encoded by *SLC22A16* [12, 13]. *ABCB1 (MDR1)* belongs to the adenosine triphosphate binding cassette family genes [14], while *SLC22A16* is a member of the organic cation transporter family [15]. Each of these transporter genes has been shown to carry genetic variations in the form of single nucleotide polymorphisms [16–18].

Since neutropenia is one of the adverse effects of DOX administration, the aim of this study was to investigate the possibility that *ABCB1 (rs10276036 C/T)* and *SLC22A16 (rs12210538 A/G)* gene polymorphisms play a role in the development of neutropenia in Iranian breast cancer patients treated with DOX-based chemotherapy.

## Methodology

### Patient information and neutropenia grading

In this case-controlled study, 100 women with breast cancer who were administered DOX-based neoadjuvant chemotherapy were selected as our study cohort. All patients were referred to the Department of Radiation Oncology of the 7-Tir Hospital in Tehran, and the presence of neutropenia was determined and graded based on their blood test report and according to the Common Terminology Criteria for Adverse Events Version 4.0. Two distinct groups were established.

The “case” group of 50 patients included those who were treated with DOX-based chemotherapy and had a neutrophil count ≤1.0 × 10^9^/L and encompassed patients with grade 3/4 febrile neutropenia, and the “control” group (50 patients) included those who were treated with DOX-based chemotherapy and had a neutrophil count >1.0 × 10^9^/L (patients with neutropenia ≤ grade 2).

### Blood collection and DNA extraction

After approval of the study by the ethics committee of the National Institute of Genetic Engineering and Biotechnology (approval code IR.NIGEB.EC.1395.5.30.A) and obtaining written informed consent from all patients, 2–3 mL of whole blood was collected into tubes containing EDTA from each; samples were stored at 4°C. Genomic DNA was extracted by using the GPP Solution Kit (Gene Pajoohan Pouya Co., Iran). Extracted DNA samples were separated on 1% agarose gels to check their quality, and were then quantified by using the UV-Vis spectrophotometer (NanoDrop 2000, Thermo Scientific, USA).

### Primer design, genotyping, and DNA sequencing

The amplification refractory mutation system-polymerase chain reaction (ARMS-PCR) method was used for genotyping the *ABCB1 (rs10276036 C/T)* and *SLC22A16 (rs12210538 A/G)* gene polymorphisms; the primers were designed using the WASP software (Genome Institute-BIOTEC, Thailand) and are shown in Table 1.

**Table 1:** Amplification refractory mutation system (ARMS) and standard-PCR primers for genotyping and sequencing of *ABCB1 (rs10276036 C/T)* and *SLC22A16 (rs12210538 A/G)* gene polymorphisms.

Method	Polymorphism	Sequence (5' → 3')	Size (bp)
ARMS-PCR	*ABCB1 (rs10276036)*	WF: CCATCAGGCTACTGAGATAGTGTCMF: CCATCAGGCTACTGAGATAGTGTTCR: GAGCCCAGGAGGTAGAGGTT	262
	*SLC22A16 (rs12210538)*	WR: CTTCGTGTGCATCGCCTTMR: CTTCGTGTGCATCGCCTCCF: TCTGCCCAAATCATACTCTGAA	215
Standard-PCR	*ABCB1 (rs10276036)*	F: TTGTGGAGAGCTGGATAAAGTGR: AGCCCAGGAGGTAGAGGTTATG	482
	*SLC22A16 (rs12210538)*	F: CCTGAACTCAAGCCATCCTCR: CTGCCTCGCAGCAATTCTTAG	581

Primers were synthesized by Macrogen, Korea. The PCR program was as follows: 1 cycle: first denaturation at 95°C for 5 min; 35 cycles: denaturation at 95°C for 1 min, annealing at 59°C for 50 s, and extension at 72°C for 50 s; and 1 cycle: final extension at 72°C for 5 min. PCR reactions were prepared according to the standard protocols [19].

**Fig 2:**
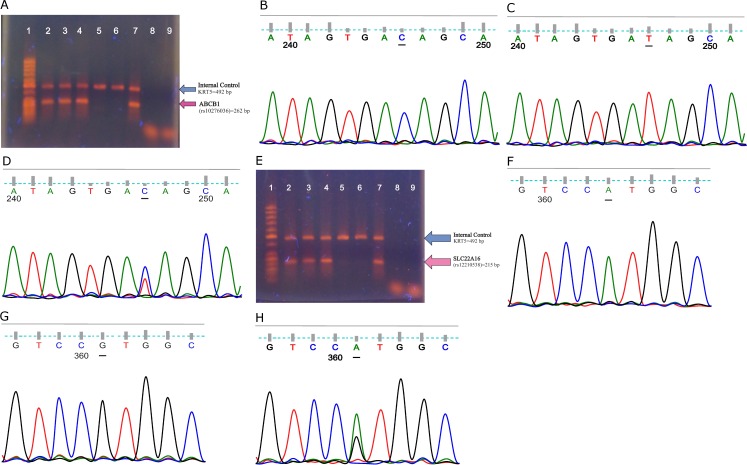
**Fig 2A. Agarose gel image of the amplification refractory mutation system**-**PCR products of the *ABCB1* gene polymorphism *(rs10276036 C/T)*.** 1: 50 bp DNA ladder, 2 & 3: heterozygote allele *C/T*, 4 & 5: homozygote allele *C*, 6 & 7: homozygote allele *T*, 8 & 9: negative control for alleles *C* and *T* respectively, **Fig 2B. Electropherogram of the *ABCB1* gene polymorphism *(rs10276036 C/T)* homozygote allele *C*, Fig 2C. Electropherogram of the *ABCB1* gene polymorphism *(rs10276036 C/T)* homozygote allele *T*, Fig 2D. Electropherogram of the *ABCB1* gene polymorphism *(rs10276036 C/T)* heterozygote allele *C/T*, Fig 2E. Agarose gel image of the amplification refractory mutation system-PCR products of the *SLC22A16* gene polymorphism *(rs12210538 A/G)*.** 1: 50 bp DNA ladder, 2 & 3: heterozygote allele *A/G*, 4 & 5: homozygote allele *A*, 6 & 7: homozygote allele *G*, 8 & 9: negative control for alleles *A* and *G* respectively, **Fig 2F. Electropherogram of the *SLC22A16* gene polymorphism *(rs12210538 A/G)* homozygote allele *A*, Fig 2G. Electropherogram of the *SLC22A16* gene polymorphism *(rs12210538 A/G)* homozygote allele *G*, Fig 2H. Electropherogram of the *SLC22A16* gene polymorphism *(rs12210538 A/G)* heterozygote allele *A/G*.**

The PCR products were then separated by electrophoresis on 1.5% agarose gels at 80–100 V for 40–50 min. The gel was stained with ethidium bromide and visualized under ultraviolet light using a full high-definition camera (Canon SX710HS, Japan). To assess the accuracy of the ARMS-PCR primers, 3 PCR products representing a wild homozygote, heterozygote, and mutant homozygote of each of the studied polymorphisms were sequenced; results were analyzed by using the NCBI Blast software (Fig 2A–2H).

### Data analysis

Statistical analysis was performed by using the IBM SPSS Statistics software ver. 23. Statistical analysis was performed on demographic, molecular, and clinical findings; the significance level was set at p<0.05.

## Results

### Distribution of neutropenia and patient demographics

In our cohort, 18% of the patients had no neutropenia; 19% had grade I, 13% had grade II, 42% had grade III, and 8% had grade IV neutropenia. Patient demographics are shown in Table 2.

**Table 2:** Demographic characteristics of the cohorts.

Character	Minimum	Maximum	Mean	St. Deviation	p
Age (y)	24	82	48.5	9.628	0.77
BMI (kg/m^2^)	18.70	51.92	28.42	5.36	0.593
W.B.C Count (M/ul)	0.5	10.4	4.176	1.5397	-

### Distribution of ethnicity and other malignancies

Fars (54%) and Turk (24%) ethnicities were the most prevalent in our cohort; based on the Pearson chi-square test, there was no significant association between ethnicity and the incidence of grade 3/4 febrile neutropenia (p = 0.191).

Most patients (70%) had no other malignancies besides breast cancer; however, 4 patients in the case group had blood hypertension, 4 had heart disease, and 4 had liver problems. On the other hand, 1 patient in the control group had arthritis and another had Parkinson’s disease. Based on the Pearson chi-square test, there was a significant association between the existence of diseases other than breast cancer and grade 3/4 febrile neutropenia (p = 0.026).

### Genotyping

The frequencies of alleles *C* and *T* in the *ABCB1* gene polymorphism *(rs10276036 C/T)* in our patients were 42.5% and 57.5%, respectively, whereas the frequencies of *CC*, *CT*, and *TT* genotypes were 28%, 29%, and 43%, respectively. The distributions were different in both patient groups; details are shown in Table 3.

**Table 3:** Allele and genotype analysis of the *ABCB1* gene polymorphism *(rs10276036 C/T)*.

Allele/Genotype	Freq. %	Dis.	OR	95% CI	RR	95% CI	p
*C*	42.5	Case: 30	1.500	0.679–3.312	1.227	0.818–1.842	0.315
		Control: 25					
*T*	57.5	Case: 31	0.515	0.217–1.223	0.733	0.500–1.075	0.130
		Control: 38					
*CC*	28	Case: 16	1.490	0.618–3.592	1.210	0.809–1.811	0.373
		Control: 12					
*CT*	29	Case: 15	1.102	0.464–2.615	1.049	0.687–1.602	0.826
		Control: 14					
*TT*	43	Case: 19	0.664	0.299–1.472	0.812	0.538–1.226	0.313
		Control: 24					

The frequencies of alleles *A* and *G* in the *SLC22A16* gene polymorphism (*rs12210538 A/G*) in the patients were 68% and 32%, respectively, whereas those of genotype *AA*, *AG*, and *GG* were 47%, 42%, and 11%, respectively. The distribution also differed between the 2 groups, as shown in Table 4.

**Table 4:** Allele and genotype analysis of the *SLC22A16* gene polymorphism *(rs12210538 A/G)*.

Allele/Genotype	Freq. %	Dis.	OR	95% CI	RR	95% CI	p
*A*	68	Case: 47	2.984	0.743–11.988	1.936	0.723–5.184	0.110
		Control: 42					
*G*	32	Case: 25	0.786	0.358–1.726	0.887	0.600–1.311	0.548
		Control: 28					
*AA*	47	Case: 25	1.273	0.579–2.795	1.128	0.763–1.668	0.548
		Control: 22					
*AG*	42	Case: 22	1.179	0.532–2.610	1.085	0.733–1.607	0.685
		Control: 20					
*GG*	11	Case: 3	0.335	0.083–1.346	0.516	0.193–1.383	0.110
		Control: 8					

### Clinical findings

In terms of cancer histology, both groups had an equal distribution of ductal carcinoma (48% each) and of lobular carcinoma (2% each).

As for cancer stages, stage IA was the least prevalent (11%), while stage IIA was the most (28%). Based on the Pearson chi-square test, there was a significant association between stage IIA (p = 0.026) and stage IIIC (p = 0.037) with grade ≤2 and grade 3/4 febrile neutropenia, respectively.

In terms of tumor grade, grades I and II were the least and most frequent at 9% and 72%, respectively.

Furthermore, the frequencies of ER, PR, and Her2 positivity were above 50% in our patients.

As for chemotherapy, ATC (adriamycin, taxotere, and cyclophosphamide) was the most frequently administered regimen (64%), whereas CAF (cyclophosphamide, adriamycin, and 5-fluorouracil) was the least commonly administered (17%). The Pearson chi-square test showed no significant association between the type of chemotherapy regimen and grade 3/4 febrile neutropenia (p = 0.917). Details are summarized in Table 5.

**Table 5:** Clinical characteristics of the patients.

Character	Freq. %	Dis.	OR	95% CI	RR	95% CI	p
Ductal Carcinoma	96	Case: 48	1.000	0.135–7.392	1.000	0.368–2.719	1.000
		Control: 48					
Lobular Carcinoma	4	Case: 2	1.000	0.135–7.392	1.000	0.368–2.719	1.000
		Control: 2					
Cancer Stage IA	11	Case: 6	1.227	0.349–4.316	1.103	0.618–1.968	0.749
		Control: 5					
Cancer Stage IIA	28	Case: 9	0.358	0.143–0.899	0.564	0.318–1.003	0.026
		Control: 19					
Cancer Stage IIB	23	Case: 10	0.712	0.279–1.818	0.837	0.501–1.398	0.476
		Control: 13					
Cancer Stage IIIA	25	Case: 15	1.714	0.683–4.301	1.286	0.861–1.920	0.248
		Control: 10					
Cancer Stage IIIC	13	Case: 10	3.917	1.008–15.220	1.673	1.150–2.434	0.037
		Control: 3					
Tumor Grade I	9	Case: 5	1.278	0.322–5.066	1.123	0.604–2.089	0.727
		Control: 4					
Tumor Grade II	72	Case: 36	1.000	0.418–2.394	1.000	0.646–1.547	1.000
		Control: 36					
Tumor Grade III	19	Case: 9	0.878	0.323–2.388	0.936	0.556–1.575	0.799
		Control: 10					
ER^+^	74	Case: 36	0.812	0.332–1.989	0.903	0.590–1.383	0.648
		Control: 38					
PR^+^	70	Case: 33	0.682	0.288–1.614	0.832	0.558–1.240	0.383
		Control: 37					
Her2^+^	52	Case: 25	0.852	0.388–1.868	0.923	0.624–1.366	0.689
		Control: 27					

## Discussion

Febrile neutropenia frequently occurs during DOX-based chemotherapy administration. Patients who experience febrile neutropenia are more likely to show poorer treatment outcomes during chemotherapy [20]; such patients often experience drug dose-related complications that lead to toxic side effects. Furthermore, these patients have longer hospitalization periods and are administered more antibiotics. It was previously reported that the incidence of febrile neutropenia in Korean breast cancer patients undergoing DOX-based chemotherapy was 29.5% [21].

In recent years, the survival rates of breast cancer patients have been improved. To further improve outcomes, the development of specific therapeutic protocols based on individuals’ genetic profiles is essential. Moreover, genetic polymorphisms were shown to affect neutrophil counts in patients who are administered DOX-based chemotherapy [22]; in these studies, the emergence of chemotherapy-induced severe toxicities was more frequent in Asian patients than in Western patients, suggesting that the former are more susceptible to febrile neutropenia.

In this study, we explored the role of *ABCB1 (rs10276036 C/T)* and *SLC22A16 (rs12210538 A/G)* gene polymorphisms in the development of grade 3/4 febrile neutropenia in Iranian breast cancer patients who were administered DOX-based chemotherapy. The *ABCB1 (rs10276036 C/T)* gene polymorphism is located in the intronic region, while the *SLC22A16 (rs12210538 A/G)* gene polymorphism is a non-synonymous polymorphism that substitutes methionine with threonine at position 409. Expression of the *SLC22A16* gene in cancer cells was associated with increased sensitivity to the cytotoxic effects of DOX [12].

Previous studies revealed that drug transporter genes such as *ABCB1* [23] and *SLC22A16* likely affect the systemic pharmacodynamics of DOX [24]. Furthermore, Bray and colleagues studied the association of polymorphic variants of the *ABCB1* and *SLC22A16* genes with drug toxicity in breast cancer patients treated with DOX, and found that polymorphisms are associated with the development of toxicities [25].

Many pharmacogenetic studies have also investigated the effect of *ABCB1* gene polymorphisms in the emergence of chemotherapy-induced side effects. In their study, Sissung and colleagues described an association between *ABCB1* gene polymorphisms and toxicities such as nephropathy, neutropenia, and survival in 23 patients who were undergoing chemotherapy [26].

In another study, it was observed that *ABCB1* and *SLC22A16* gene polymorphisms were associated with drug-induced side effects such as leukopenia, although they had no effect on survival [13]. Furthermore, it has been demonstrated that the *ABCB1* variant 13435 *C/T* is associated with greater mRNA instability, which can lead to more prevalent grade 3/4 febrile neutropenia [23].

Even though our study revealed no significant association between *ABCB1 (rs10276036 C/T)* and *SLC22A16 (rs12210538 A/G)* gene polymorphisms and grade 3/4 febrile neutropenia, *ABCB1 (rs10276036 C/T)* allele C polymorphisms (genotypes CC and CT) and *SLC22A16 (rs12210538 A/G)* allele A polymorphisms (genotypes AA and AG) tended to be associated with more frequent grade 3/4 febrile neutropenia. On the other hand, the *ABCB1 (rs10276036 C/T)* allele T polymorphism (genotype TT) and the *SLC22A16 (rs12210538 A/G)* allele G polymorphism (genotype GG) appeared to have a protective role against grade 3/4 febrile neutropenia.

## Conclusions

Our results show that carriers of at least one allele *C* of *ABCB1 (rs10276036 C/T)* polymorphism and at least one allele *A* of *SLC22A16 (rs12210538 A/G)* polymorphism tend to be more susceptible to grade 3/4 febrile neutropenia. Therefore, a combination of additional molecular and clinical tests ought to be useful for evaluating the risk of grade 3/4 febrile neutropenia.

This study included limitations such as the fact that not all genes related to DOX metabolism and transport were investigated, and that the results are based on examining patients who underwent DOX-based combination chemotherapy and not DOX alone. Furthermore, the study included a small cohort size, and all patients were from a single clinical center, where administration of prophylactic granulocyte-colony stimulating factor and of overall drug doses was at the physicians’ discretion. To validate the results of this study, further investigations with larger cohort sizes in various populations are required to further explore the effect of these polymorphisms in the development of grade 3/4 febrile neutropenia.

## Supporting Information

S1 File ABCB1-Crosstabs. (PDF)

S2 File SLC22A16-Crosstabs. (PDF)
