# Supplementary material for: Association of ABCB1 and SLC22A16 Gene Polymorphisms with Incidence of Doxorubicin-Induced Febrile Neutropenia: A Survey of Iranian Breast Cancer Patients
Source: PLoS One. 2016 Dec 30;11(12):e0168519. doi: 10.1371/journal.pone.0168519 (PMC5201260; doi:10.1371/journal.pone.0168519)
Supplement: S2 File — (PDF) [file pone.0168519.s002.pdf]

```
 DATASET CLOSE DataSet3.
CROSSTABS
  /TABLES=AlleleA AlleleG GenotypeAA GenotypeAG GenotypeGG Age Weight Height E
thnicity W.B.C Normal
    Grade1 Grade2 Grade3 Grade4 ER PR HER2 Regimen etc Ductal Lobular StageIA
StageIIA StageIIB
    StageIIIA StageIIIC GradeI GradeII GradeIII BY Groups
  /FORMAT=AVALUE TABLES
  /STATISTICS=CHISQ RISK
  /CELLS=COUNT
  /COUNT ROUND CELL
  /BARCHART.
```

Crosstabs

Notes

Output Created		16-SEP-2016 13:00:51
Comments		
Input	Data	C:\Users\Maryam\Desktop\SPSS-Case-Control\SLC22A16.sav
	Active Dataset	DataSet4
	Filter	<none>
	Weight	<none>
	Split File	<none>
	N of Rows in Working Data File	100
Missing Value Handling	Definition of Missing	User-defined missing values are treated as missing.
	Cases Used	Statistics for each table are based on all the cases with valid data in the specified range(s) for all variables in each table.

Notes

Syntax		CROSSTABS<br>/TABLES=AlleleA AlleleG GenotypeAA GenotypeAG GenotypeGG Age Weight Height Ethnicity W.B.C Normal<br>Grade1 Grade2 Grade3 Grade4 ER PR HER2 Regimen etc Ductal Lobular StageIA StageIIA StageIIB<br>StageIIIA StageIIIC GradeI GradeII GradeIII BY Groups<br>/FORMAT=AVALUE TABLES<br>/STATISTICS=CHISQ RISK<br>/CELLS=COUNT<br>/COUNT ROUND CELL<br>/BARCHART.
Resources	Processor Time	00:00:04.59
	Elapsed Time	00:00:04.15
	Dimensions Requested	2
	Cells Available	524245

Case Processing Summary

	Cases					
	Valid		Missing		Total	
	N	Percent	N	Percent	N	Percent
SLC22A16 Allele A * Pt. Groups	100	100.0%	0	0.0%	100	100.0%
SLC22A16 Allele G * Pt. Groups	100	100.0%	0	0.0%	100	100.0%
SLC22A16 Genotypes Genotype AA * Pt. Groups	100	100.0%	0	0.0%	100	100.0%
SLC22A16 Genotypes Genotype AG * Pt. Groups	100	100.0%	0	0.0%	100	100.0%
SLC22A16 Genotypes Genotype GG * Pt. Groups	100	100.0%	0	0.0%	100	100.0%
Pt. Age * Pt. Groups	100	100.0%	0	0.0%	100	100.0%
Pt. Weight * Pt. Groups	100	100.0%	0	0.0%	100	100.0%
Pt. Height * Pt. Groups	100	100.0%	0	0.0%	100	100.0%
Pt. Ethnicity * Pt. Groups	100	100.0%	0	0.0%	100	100.0%
Pt. WBC Count * Pt. Groups	100	100.0%	0	0.0%	100	100.0%
Pt. Without Neutropenia * Pt. Groups	100	100.0%	0	0.0%	100	100.0%

Case Processing Summary

	Cases					
	Valid		Missing		Total	
	N	Percent	N	Percent	N	Percent
Pt. Neutropenia Grade 1 * Pt. Groups	100	100.0%	0	0.0%	100	100.0%
Pt. Neutropenia Grade 2 * Pt. Groups	100	100.0%	0	0.0%	100	100.0%
Pt. Neutropenia Grade 3 * Pt. Groups	100	100.0%	0	0.0%	100	100.0%
Pt. Neutropenia Grade 4 * Pt. Groups	100	100.0%	0	0.0%	100	100.0%
Estrogen Receptor * Pt. Groups	100	100.0%	0	0.0%	100	100.0%
Progesterone Receptor * Pt. Groups	100	100.0%	0	0.0%	100	100.0%
Human Epidermal Growth Factor Receptor 2 * Pt. Groups	100	100.0%	0	0.0%	100	100.0%
Chemotherapy Regimen * Pt. Groups	100	100.0%	0	0.0%	100	100.0%
Other Disease * Pt. Groups	100	100.0%	0	0.0%	100	100.0%
Ductal Carcinoma * Pt. Groups	100	100.0%	0	0.0%	100	100.0%
Lobular Carcinoma * Pt. Groups	100	100.0%	0	0.0%	100	100.0%
Cancer Stage IA * Pt. Groups	100	100.0%	0	0.0%	100	100.0%
Cancer Stage IIA * Pt. Groups	100	100.0%	0	0.0%	100	100.0%
Cancer Stage IIB * Pt. Groups	100	100.0%	0	0.0%	100	100.0%
Cancer Stage IIIA * Pt. Groups	100	100.0%	0	0.0%	100	100.0%
Cancer Stage IIIC * Pt. Groups	100	100.0%	0	0.0%	100	100.0%
Tumor Grade I * Pt. Groups	100	100.0%	0	0.0%	100	100.0%
Tumor Grade II * Pt. Groups	100	100.0%	0	0.0%	100	100.0%
Tumor Grade III * Pt. Groups	100	100.0%	0	0.0%	100	100.0%

SLC22A16 Allele A * Pt. Groups

Crosstab

Count

		Pt. Groups		Total
		Case	Control	
SLC22A16 Allele A	Yes	47	42	89
	No	3	8	11
Total		50	50	100

Chi-Square Tests

	Value	df	Asymptotic Significance (2-sided)	Exact Sig. (2-sided)	Exact Sig. (1-sided)
Pearson Chi-Square	2.554[a]	1	.110		
Continuity Correction[b]	1.634	1	.201		
Likelihood Ratio	2.639	1	.104		
Fisher's Exact Test				.200	.100
N of Valid Cases	100				

a. 0 cells (0.0%) have expected count less than 5. The minimum expected count is 5.50.

b. Computed only for a 2x2 table

Risk Estimate

	Value	95% Confidence Interval	
		Lower	Upper
Odds Ratio for SLC22A16 Allele A (Yes / No)	2.984	.743	11.988
For cohort Pt. Groups = Case	1.936	.723	5.184
For cohort Pt. Groups = Control	.649	.425	.991
N of Valid Cases	100		

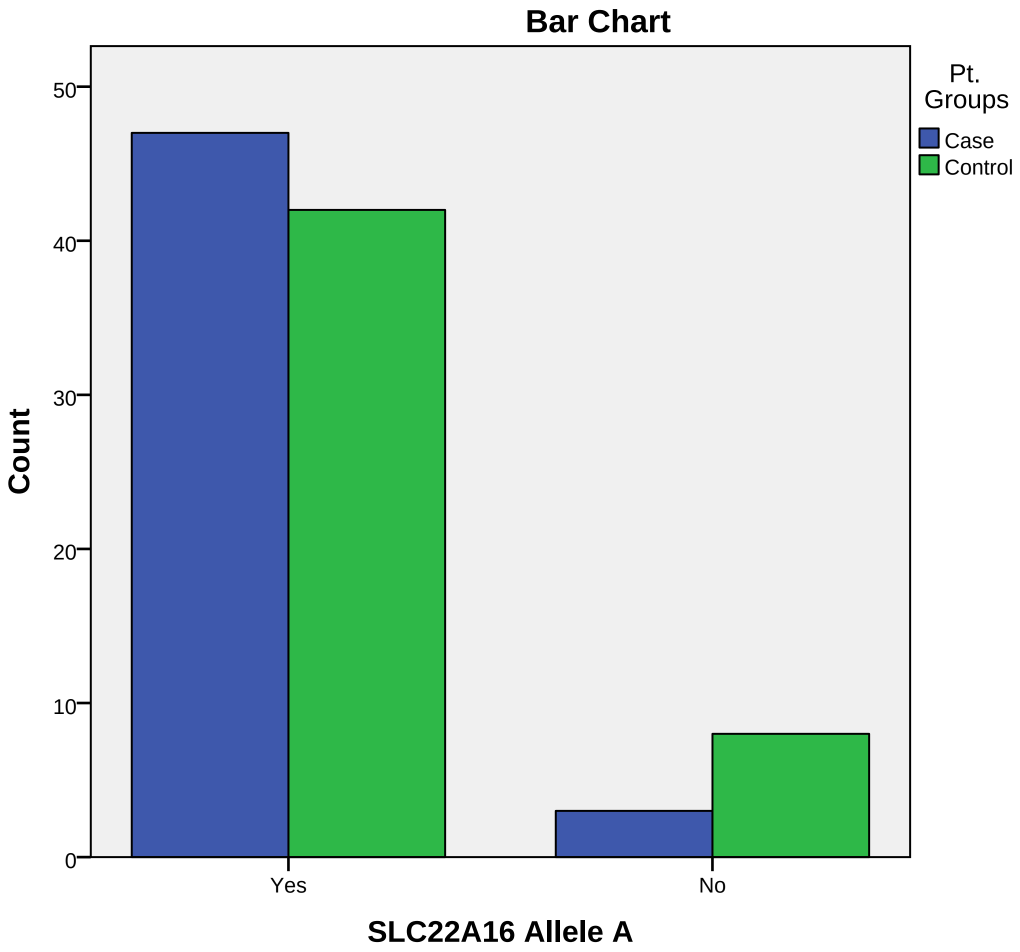

SLC22A16 Allele G * Pt. Groups

Crosstab

Count

		Pt. Groups		Total
		Case	Control	
SLC22A16 Allele G	Yes	25	28	53
	No	25	22	47
Total		50	50	100

Chi-Square Tests

	Value	df	Asymptotic Significance (2-sided)	Exact Sig. (2-sided)	Exact Sig. (1-sided)
Pearson Chi-Square	.361[a]	1	.548		
Continuity Correction[b]	.161	1	.689		
Likelihood Ratio	.362	1	.548		
Fisher's Exact Test				.689	.344
N of Valid Cases	100				

a. 0 cells (0.0%) have expected count less than 5. The minimum expected count is 23.50.

b. Computed only for a 2x2 table

Risk Estimate

	Value	95% Confidence Interval	
		Lower	Upper
Odds Ratio for SLC22A16 Allele G (Yes / No)	.786	.358	1.726
For cohort Pt. Groups = Case	.887	.600	1.311
For cohort Pt. Groups = Control	1.129	.759	1.679
N of Valid Cases	100		

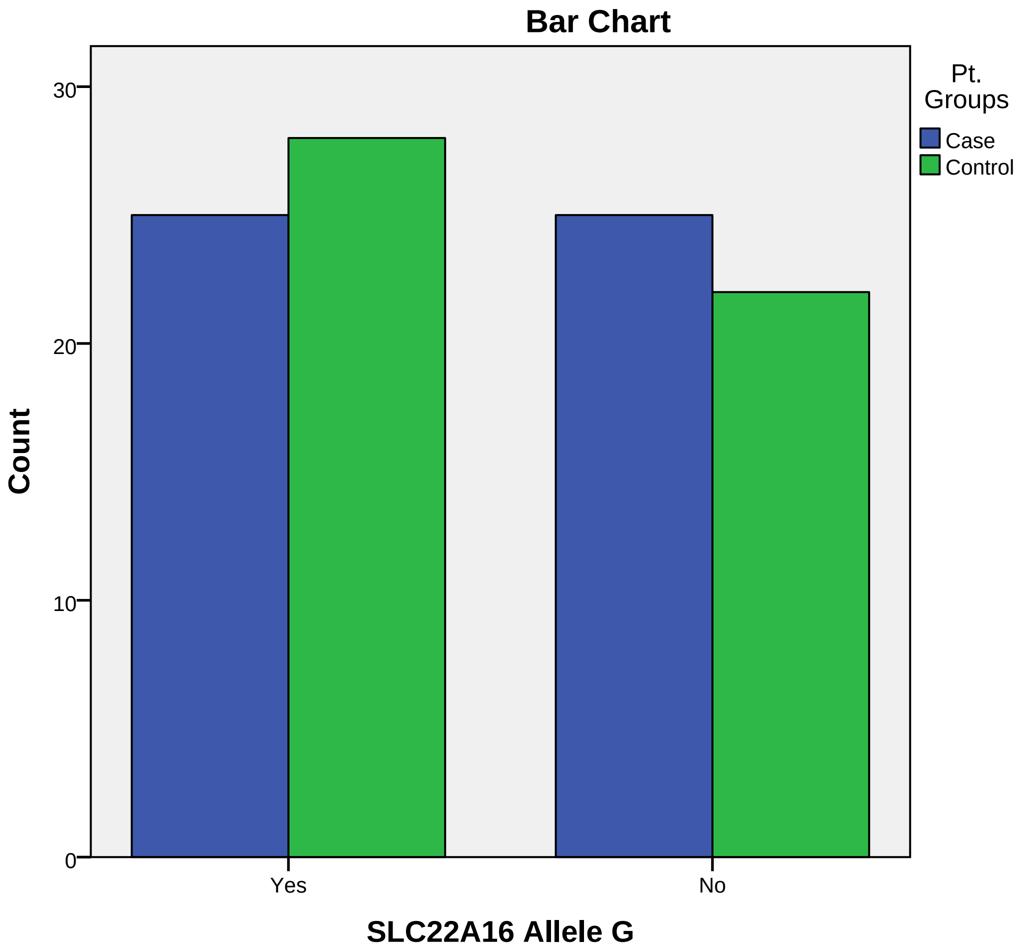

SLC22A16 Genotypes Genotype AA * Pt. Groups

Crosstab

Count

		Pt. Groups		Total
		Case	Control	
SLC22A16 Genotypes Genotype AA	Yes	25	22	47
	No	25	28	53
Total		50	50	100

Chi-Square Tests

	Value	df	Asymptotic Significance (2-sided)	Exact Sig. (2-sided)	Exact Sig. (1-sided)
Pearson Chi-Square	.361[a]	1	.548		
Continuity Correction[b]	.161	1	.689		
Likelihood Ratio	.362	1	.548		
Fisher's Exact Test				.689	.344
N of Valid Cases	100				

a. 0 cells (0.0%) have expected count less than 5. The minimum expected count is 23.50.

b. Computed only for a 2x2 table

Risk Estimate

	Value	95% Confidence Interval	
		Lower	Upper
Odds Ratio for SLC22A16 Genotypes Genotype AA (Yes / No)	1.273	.579	2.795
For cohort Pt. Groups = Case	1.128	.763	1.668
For cohort Pt. Groups = Control	.886	.596	1.318
N of Valid Cases	100		

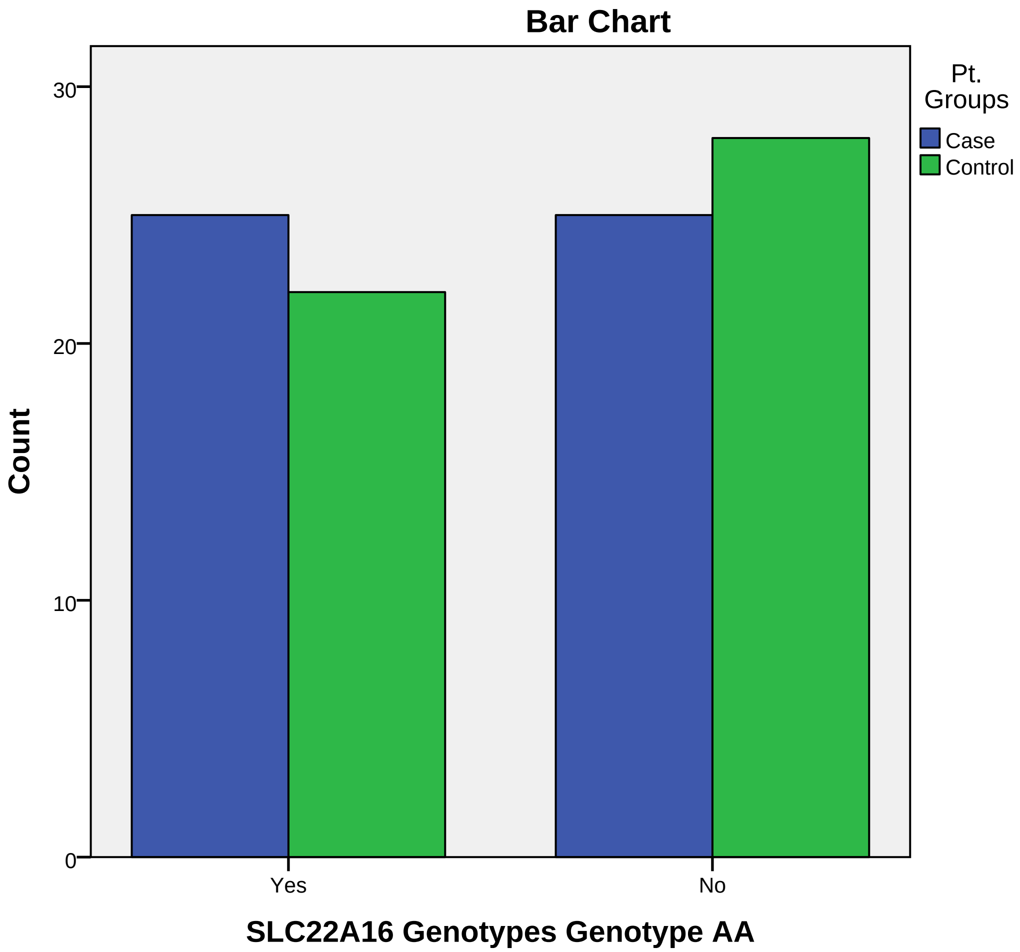

SLC22A16 Genotypes Genotype AG * Pt. Groups

Crosstab

Count

		Pt. Groups		Total
		Case	Control	
SLC22A16 Genotypes Genotype AG	Yes	22	20	42
	No	28	30	58
Total		50	50	100

Chi-Square Tests

	Value	df	Asymptotic Significance (2-sided)	Exact Sig. (2-sided)	Exact Sig. (1-sided)
Pearson Chi-Square	.164[a]	1	.685		
Continuity Correction[b]	.041	1	.839		
Likelihood Ratio	.164	1	.685		
Fisher's Exact Test				.840	.420
N of Valid Cases	100				

a. 0 cells (0.0%) have expected count less than 5. The minimum expected count is 21.00.

b. Computed only for a 2x2 table

Risk Estimate

	Value	95% Confidence Interval	
		Lower	Upper
Odds Ratio for SLC22A16 Genotypes Genotype AG (Yes / No)	1.179	.532	2.610
For cohort Pt. Groups = Case	1.085	.733	1.607
For cohort Pt. Groups = Control	.921	.615	1.378
N of Valid Cases	100		

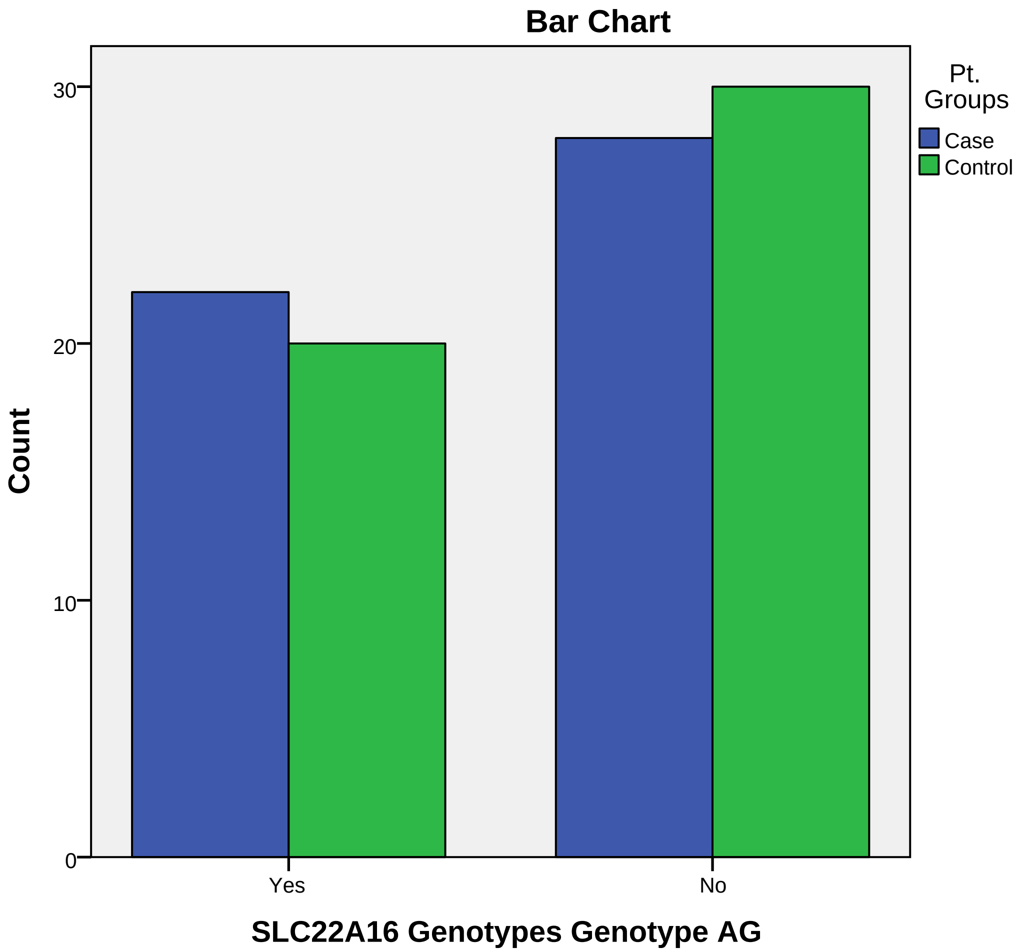

SLC22A16 Genotypes Genotype GG * Pt. Groups

Crosstab

Count

		Pt. Groups		Total
		Case	Control	
SLC22A16 Genotypes Genotype GG	Yes	3	8	11
	No	47	42	89
Total		50	50	100

Chi-Square Tests

	Value	df	Asymptotic Significance (2-sided)	Exact Sig. (2-sided)	Exact Sig. (1-sided)
Pearson Chi-Square	2.554[a]	1	.110		
Continuity Correction[b]	1.634	1	.201		
Likelihood Ratio	2.639	1	.104		
Fisher's Exact Test				.200	.100
N of Valid Cases	100				

a. 0 cells (0.0%) have expected count less than 5. The minimum expected count is 5.50.

b. Computed only for a 2x2 table

Risk Estimate

	Value	95% Confidence Interval	
		Lower	Upper
Odds Ratio for SLC22A16 Genotypes Genotype GG (Yes / No)	.335	.083	1.346
For cohort Pt. Groups = Case	.516	.193	1.383
For cohort Pt. Groups = Control	1.541	1.009	2.354
N of Valid Cases	100		

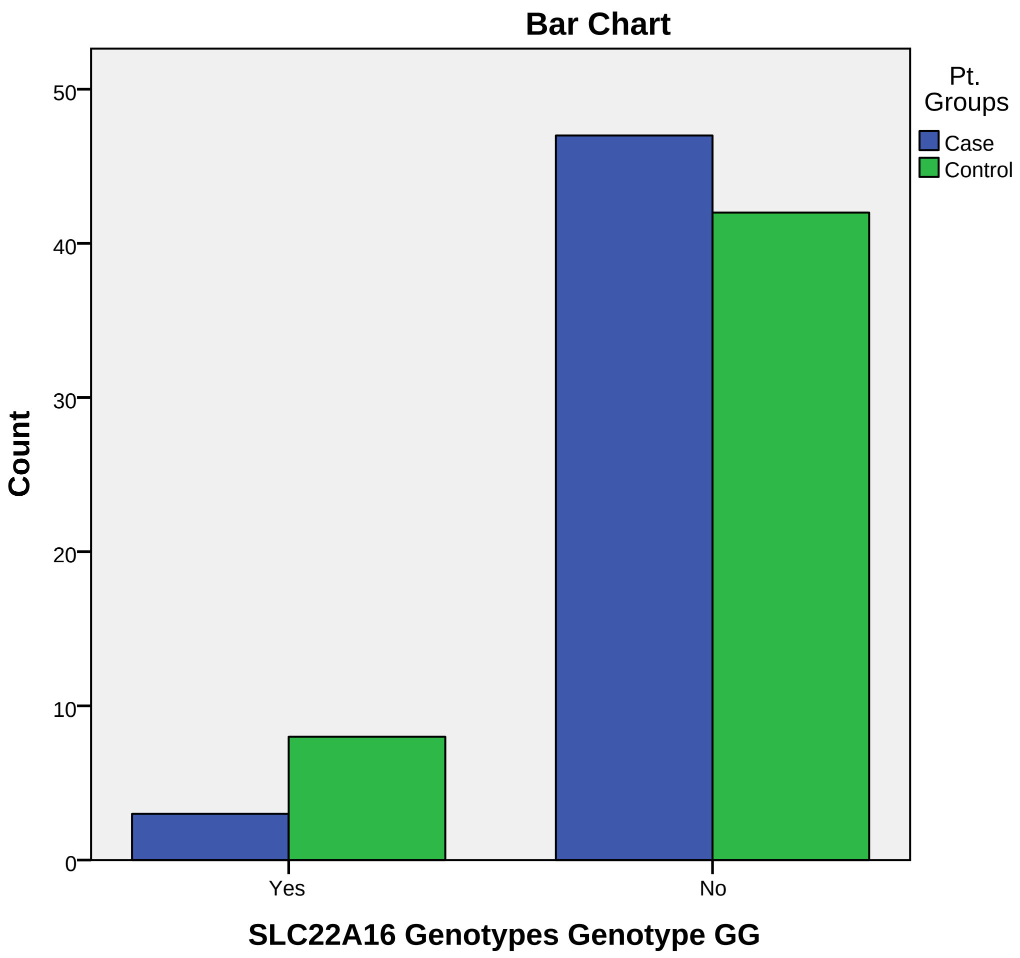

Pt. Age * Pt. Groups

Crosstab

Count

		Pt. Groups		
		Case	Control	Total
Pt. Age	24	1	0	1
	30	0	1	1
	31	1	1	2
	32	0	1	1
	33	1	0	1
	35	1	0	1
	36	1	1	2
	37	1	1	2
	38	2	2	4
	39	1	2	3
	40	1	3	4
	41	1	0	1
	42	1	4	5
	43	3	1	4
	44	1	1	2
	45	3	1	4
	46	1	3	4
	47	2	0	2
	48	5	3	8
	49	0	1	1
	50	4	4	8
	51	1	1	2
	52	2	2	4
	53	1	2	3
	54	2	2	4
	55	3	2	5
	56	3	0	3
	57	0	3	3
	58	0	1	1
	60	1	2	3
	61	1	0	1
	62	0	1	1
	63	3	1	4
	65	1	1	2
	66	1	0	1
	67	0	1	1
	82	0	1	1

Crosstab

Count

	Pt. Groups		Total
	Case	Control	
Total	50	50	100

Chi-Square Tests

	Value	df	Asymptotic Significance (2-sided)
Pearson Chi-Square	29.500[a]	36	.770
Likelihood Ratio	37.998	36	.378
N of Valid Cases	100		

a. 74 cells (100.0%) have expected count less than 5. The minimum expected count is .50.

Risk Estimate

	Value
Odds Ratio for Pt. Age (24 / 30)	a

a. Risk Estimate statistics cannot be computed. They are only computed for a 2*2 table without empty cells.

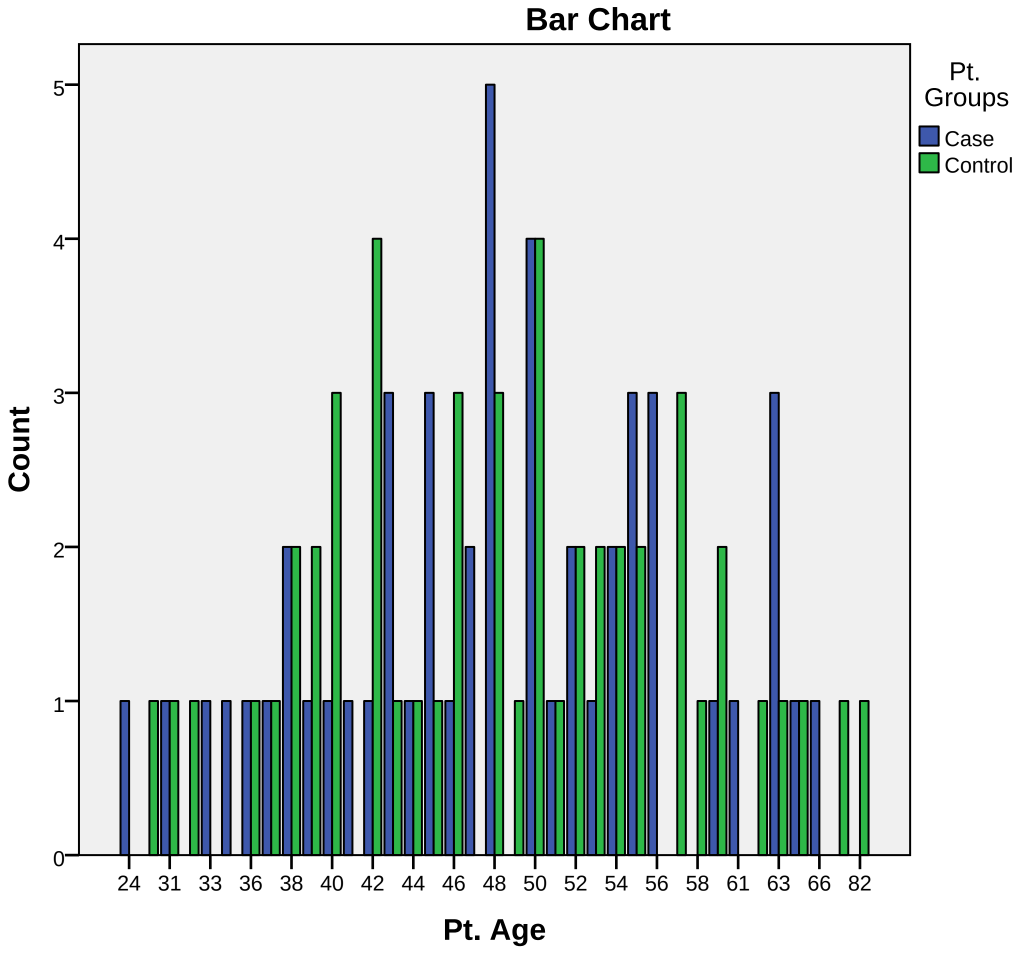

Pt. Weight * Pt. Groups

Crosstab

Count

		Pt. Groups		Total
		Case	Control	
Pt. Weight	48	0	1	1
	50	1	0	1

Crosstab

Count

	Pt. Groups		Total
	Case	Control	
51	1	0	1
52	1	1	2
54	1	1	2
55	2	0	2
57	1	1	2
58	0	1	1
60	4	2	6
62	2	2	4
63	1	2	3
65	1	2	3
66	3	0	3
67	1	1	2
68	1	3	4
69	1	1	2
70	3	2	5
71	1	2	3
72	4	1	5
73	1	1	2
74	0	4	4
75	7	3	10
77	0	2	2
78	2	1	3
80	1	3	4
81	1	1	2
82	5	1	6
83	0	1	1
84	0	1	1
85	3	2	5
86	0	1	1
88	0	1	1
92	1	0	1
96	0	1	1
100	0	1	1
104	0	1	1
110	0	1	1
148	0	1	1
Total	50	50	100

Chi-Square Tests

	Value	df	Asymptotic Significance (2-sided)
Pearson Chi-Square	35.467[a]	37	.541
Likelihood Ratio	45.676	37	.155
N of Valid Cases	100		

a. 74 cells (97.4%) have expected count less than 5. The minimum expected count is .50.

Risk Estimate

	Value
Odds Ratio for Pt. Weight (48 / 50)	[a]

a. Risk Estimate statistics cannot be computed. They are only computed for a 2*2 table without empty cells.

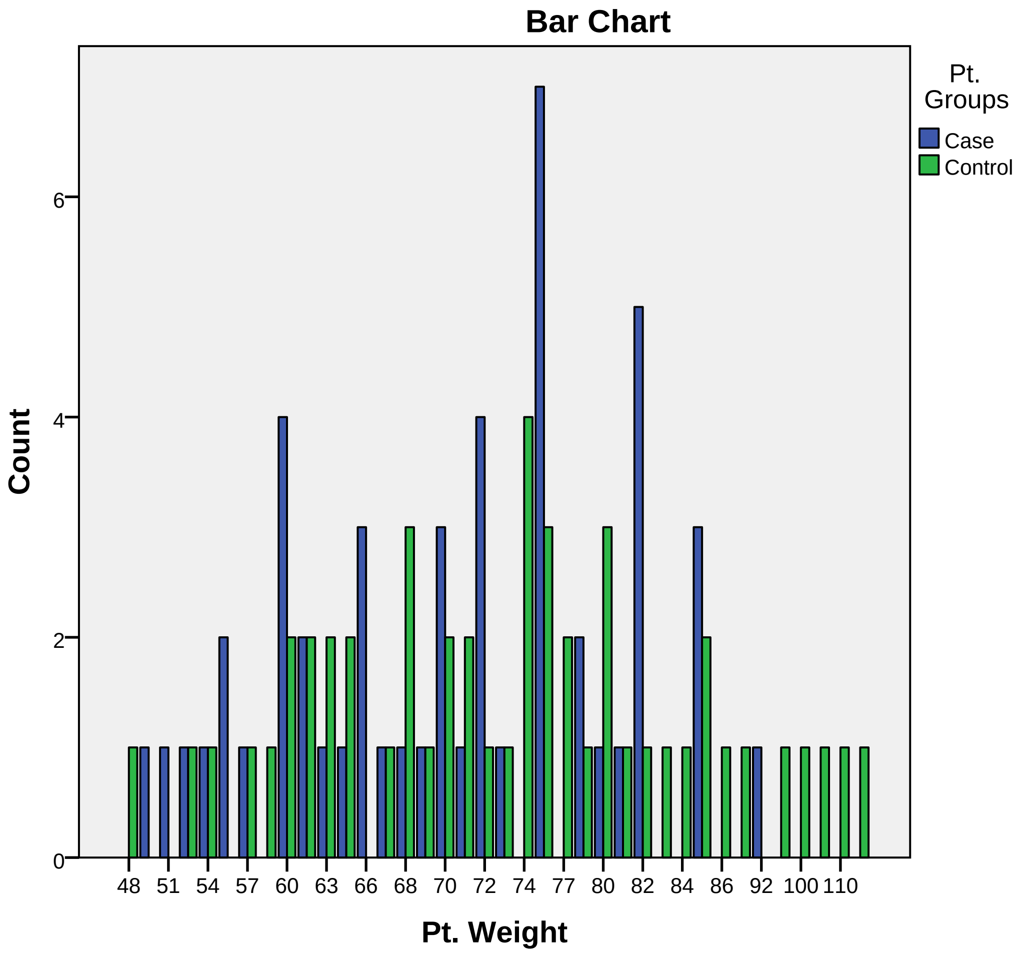

Pt. Height * Pt. Groups

Crosstab

Count

		Pt. Groups		
		Case	Control	Total
Pt. Height	140	1	0	1
	145	1	0	1
	148	0	1	1
	150	2	7	9
	152	1	1	2
	153	0	2	2
	155	7	2	9
	156	2	1	3
	157	2	2	4
	158	5	2	7
	159	2	1	3
	160	5	13	18
	161	2	0	2
	162	2	2	4
	163	3	1	4
	164	1	1	2
	165	4	4	8
	166	2	1	3
	167	2	2	4
	168	0	1	1
	169	0	2	2
	170	4	2	6
	175	2	1	3
	180	0	1	1
Total		50	50	100

Chi-Square Tests

	Value	df	Asymptotic Significance (2-sided)
Pearson Chi-Square	24.397[a]	23	.382
Likelihood Ratio	29.230	23	.173
N of Valid Cases	100		

a. 46 cells (95.8%) have expected count less than 5. The minimum expected count is .50.

Risk Estimate

	Value
Odds Ratio for Pt. Height (140 / 145)	a

a. Risk Estimate statistics cannot be computed. They are only computed for a 2*2 table without empty cells.

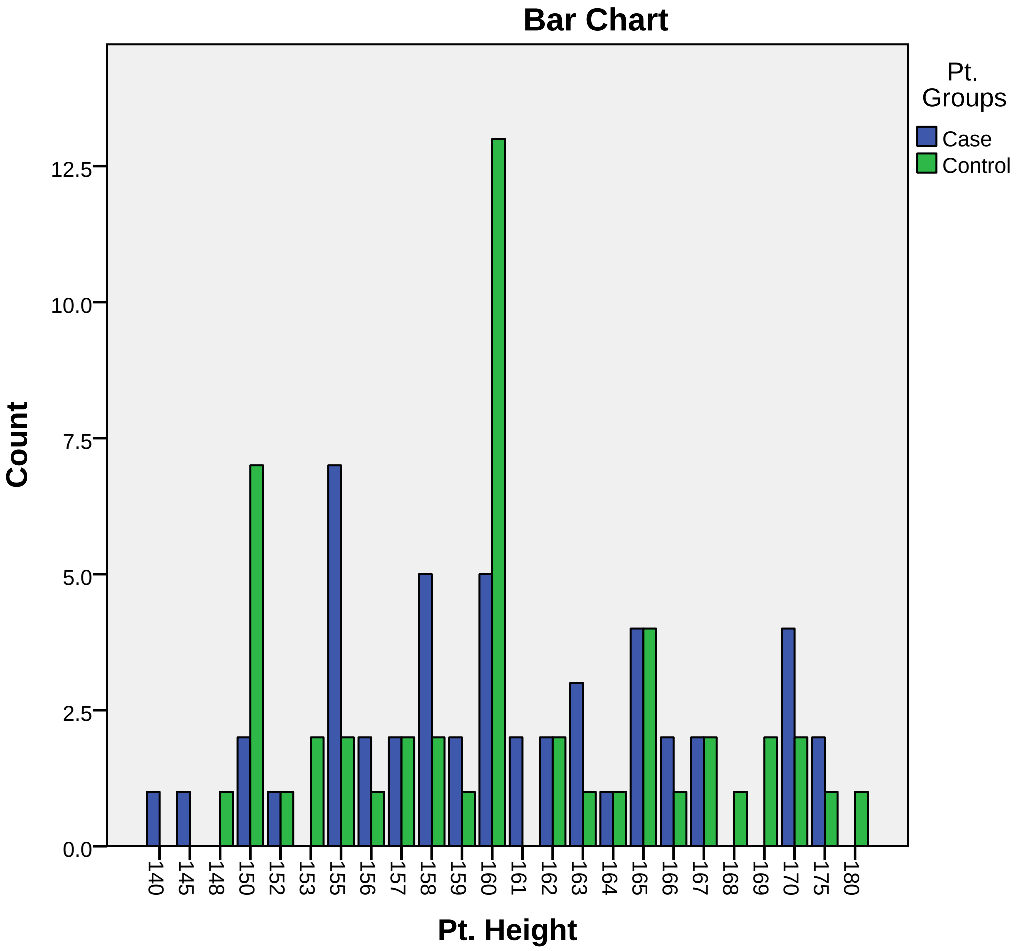

Pt. Ethnicity * Pt. Groups

Crosstab

Count

		Pt. Groups		Total
		Case	Control	
Pt. Ethnicity	Fars	25	29	54
	Afghan	3	1	4
	Tork	12	14	26
	Lor	0	2	2
	Kord	7	1	8
	Arab	0	1	1
	Gilak	2	2	4
	Mazani	1	0	1
Total		50	50	100

Chi-Square Tests

	Value	df	Asymptotic Significance (2-sided)
Pearson Chi-Square	9.950[a]	7	.191
Likelihood Ratio	12.104	7	.097
N of Valid Cases	100		

a. 12 cells (75.0%) have expected count less than 5. The minimum expected count is .50.

Risk Estimate

	Value
Odds Ratio for Pt. Ethnicity (Fars / Afghan)	[a]

a. Risk Estimate statistics cannot be computed. They are only computed for a 2*2 table without empty cells.

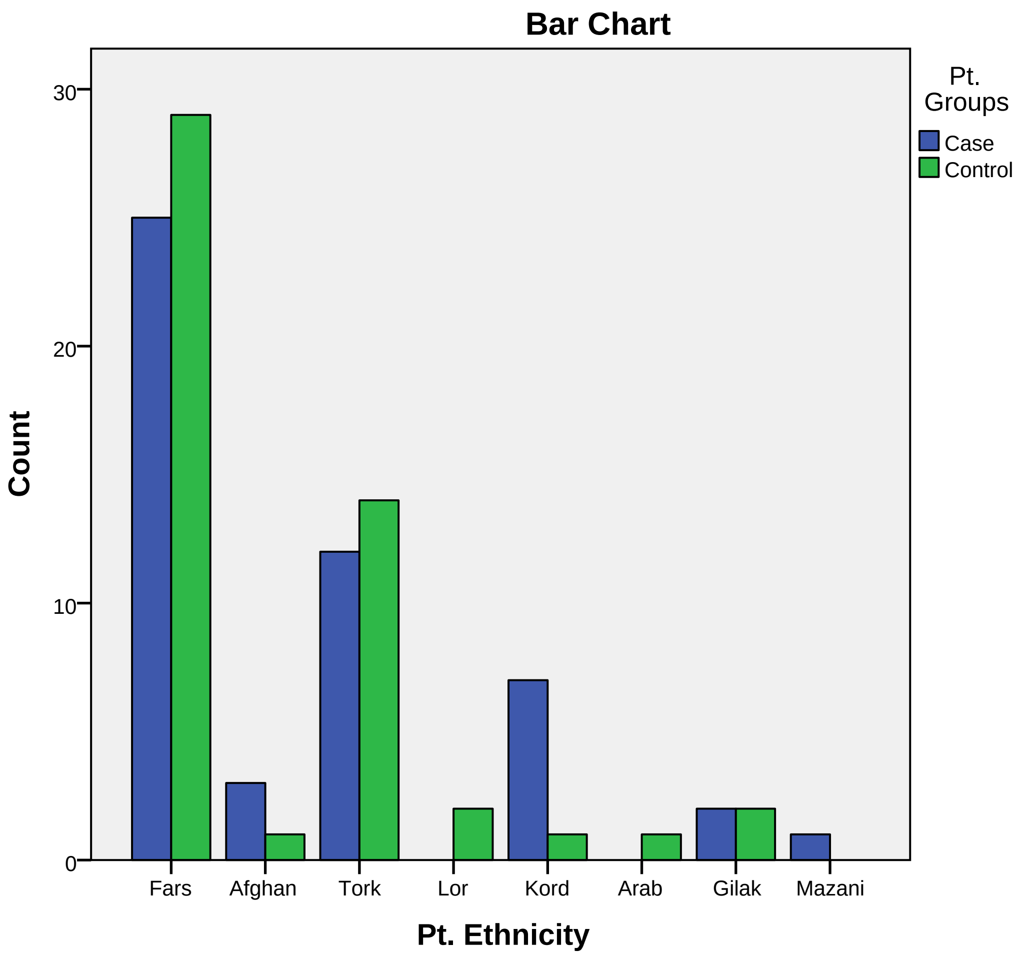

Pt. WBC Count * Pt. Groups

Crosstab

Count

		Pt. Groups		
		Case	Control	Total
Pt. WBC Count	.5	1	0	1
	.8	1	0	1
	.9	1	0	1
	1.2	1	0	1
	2.1	1	0	1
	2.3	3	0	3
	2.6	6	0	6
	2.8	1	0	1
	2.9	1	0	1
	3.0	3	0	3
	3.1	2	0	2
	3.2	2	0	2
	3.3	1	0	1
	3.4	4	0	4
	3.5	22	0	22
	4.2	0	2	2
	4.3	0	4	4
	4.4	0	5	5
	4.5	0	2	2
	4.6	0	3	3
	4.7	0	2	2
	4.8	0	2	2
	4.9	0	3	3
	5.0	0	1	1
	5.1	0	3	3
	5.2	0	4	4
	5.3	0	1	1
	5.6	0	1	1
	5.7	0	2	2
	5.8	0	2	2
	5.9	0	1	1
	6.0	0	1	1
	6.3	0	3	3
	6.4	0	1	1
	6.5	0	1	1
	6.7	0	1	1
	6.8	0	1	1

Crosstab

Count

	Pt. Groups		
	Case	Control	Total
6.9	0	1	1
7.0	0	1	1
7.8	0	1	1
10.4	0	1	1
Total	50	50	100

Chi-Square Tests

	Value	df	Asymptotic Significance (2-sided)
Pearson Chi-Square	100.000[a]	40	.000
Likelihood Ratio	138.629	40	.000
N of Valid Cases	100		

a. 80 cells (97.6%) have expected count less than 5. The minimum expected count is .50.

Risk Estimate

	Value
Odds Ratio for Pt. WBC Count (.5 / .8)	[a]

a. Risk Estimate statistics cannot be computed. They are only computed for a 2*2 table without empty cells.

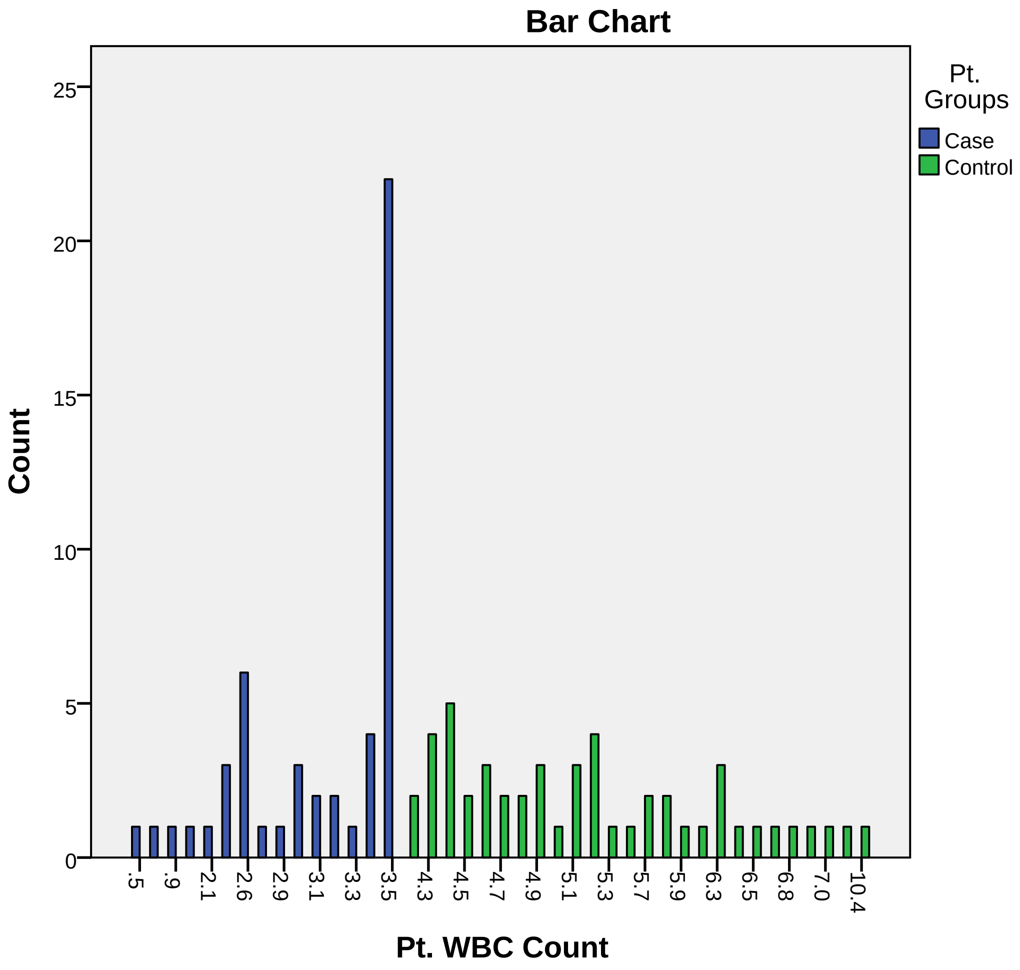

Pt. Without Neutropenia * Pt. Groups

Crosstab

Count

		Pt. Groups		Total
		Case	Control	
Pt. Without Neutropenia	Yes	0	17	17
	No	50	33	83
Total		50	50	100

Chi-Square Tests

	Value	df	Asymptotic Significance (2-sided)	Exact Sig. (2-sided)	Exact Sig. (1-sided)
Pearson Chi-Square	20.482[a]	1	.000		
Continuity Correction[b]	18.143	1	.000		
Likelihood Ratio	27.074	1	.000		
Fisher's Exact Test				.000	.000
N of Valid Cases	100				

a. 0 cells (0.0%) have expected count less than 5. The minimum expected count is 8.50.

b. Computed only for a 2x2 table

Risk Estimate

	Value	95% Confidence Interval	
		Lower	Upper
For cohort Pt. Groups = Control	2.515	1.930	3.278
N of Valid Cases	100		

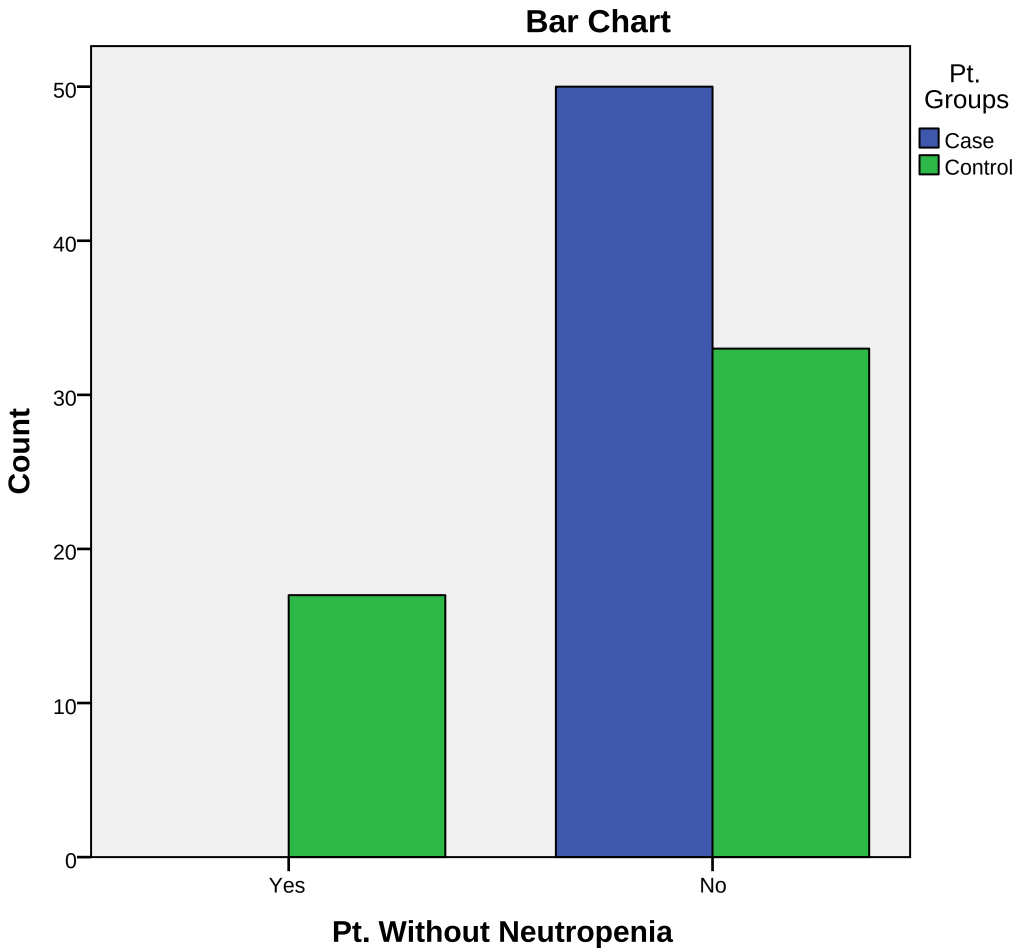

Pt. Neutropenia Grade 1 * Pt. Groups

Crosstab

Count

		Pt. Groups		Total
		Case	Control	
Pt. Neutropenia Grade 1	Yes	0	2	2
	No	50	48	98
Total		50	50	100

Chi-Square Tests

	Value	df	Asymptotic Significance (2-sided)	Exact Sig. (2-sided)	Exact Sig. (1-sided)
Pearson Chi-Square	2.041[a]	1	.153		
Continuity Correction[b]	.510	1	.475		
Likelihood Ratio	2.813	1	.093		
Fisher's Exact Test				.495	.247
N of Valid Cases	100				

a. 2 cells (50.0%) have expected count less than 5. The minimum expected count is 1.00.

b. Computed only for a 2x2 table

Risk Estimate

	Value	95% Confidence Interval	
		Lower	Upper
For cohort Pt. Groups = Control	2.042	1.668	2.499
N of Valid Cases	100		

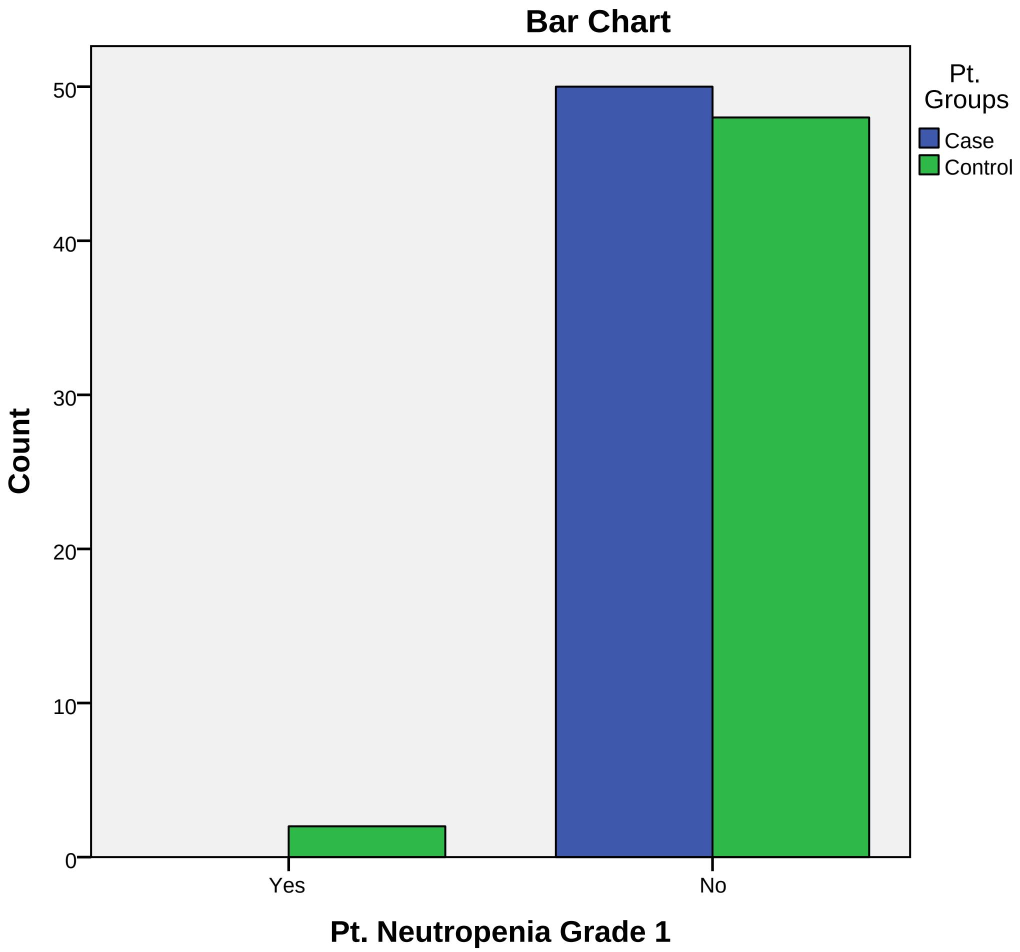

Pt. Neutropenia Grade 2 * Pt. Groups

Crosstab

Count

		Pt. Groups		Total
		Case	Control	
Pt. Neutropenia Grade 2	Yes	0	30	30
	No	50	20	70
Total		50	50	100

Chi-Square Tests

	Value	df	Asymptotic Significance (2-sided)	Exact Sig. (2-sided)	Exact Sig. (1-sided)
Pearson Chi-Square	42.857[a]	1	.000		
Continuity Correction[b]	40.048	1	.000		
Likelihood Ratio	54.872	1	.000		
Fisher's Exact Test				.000	.000
N of Valid Cases	100				

a. 0 cells (0.0%) have expected count less than 5. The minimum expected count is 15.00.

b. Computed only for a 2x2 table

Risk Estimate

	Value	95% Confidence Interval	
		Lower	Upper
For cohort Pt. Groups = Control	3.500	2.417	5.069
N of Valid Cases	100		

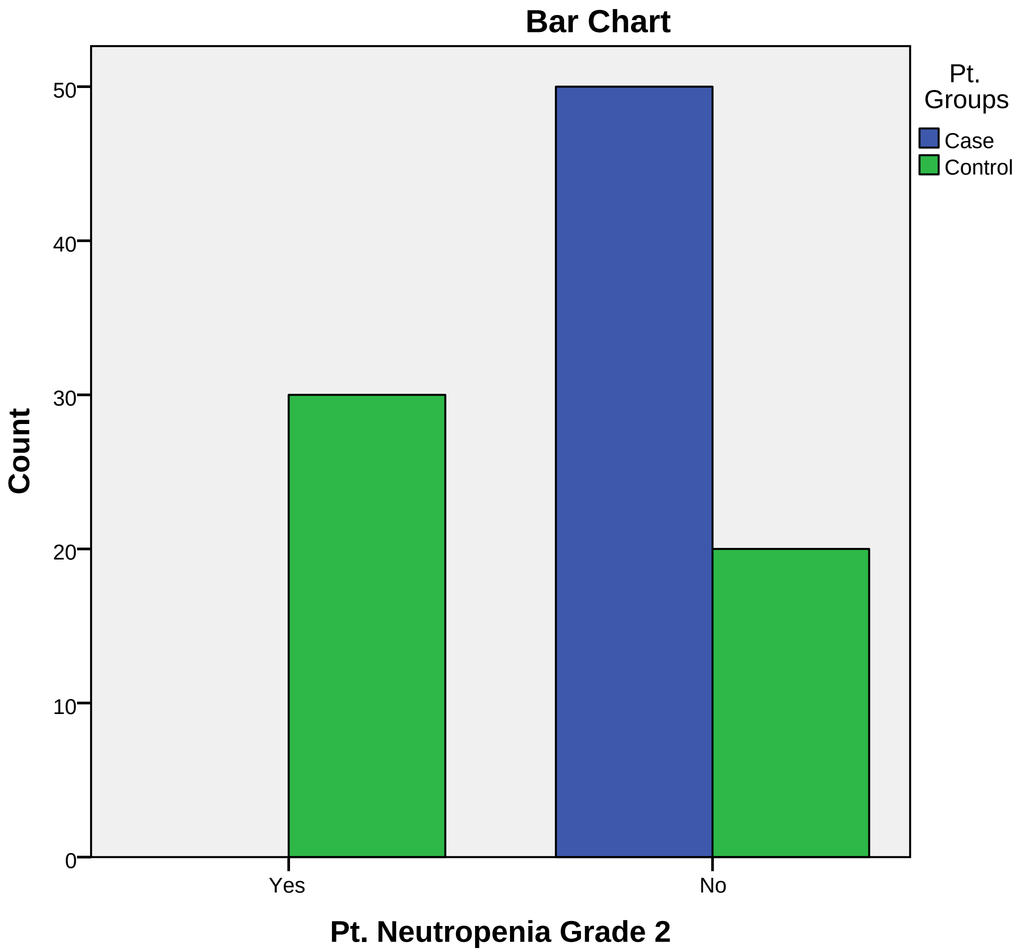

Pt. Neutropenia Grade 3 * Pt. Groups

Crosstab

Count

		Pt. Groups		
		Case	Control	Total
Pt. Neutropenia Grade 3	Yes	42	0	42
	No	8	50	58
Total		50	50	100

Chi-Square Tests

	Value	df	Asymptotic Significance (2-sided)	Exact Sig. (2-sided)	Exact Sig. (1-sided)
Pearson Chi-Square	72.414[a]	1	.000		
Continuity Correction[b]	69.007	1	.000		
Likelihood Ratio	92.091	1	.000		
Fisher's Exact Test				.000	.000
N of Valid Cases	100				

a. 0 cells (0.0%) have expected count less than 5. The minimum expected count is 21.00.

b. Computed only for a 2x2 table

Risk Estimate

	Value	95% Confidence Interval	
		Lower	Upper
For cohort Pt. Groups = Case	7.250	3.810	13.796
N of Valid Cases	100		

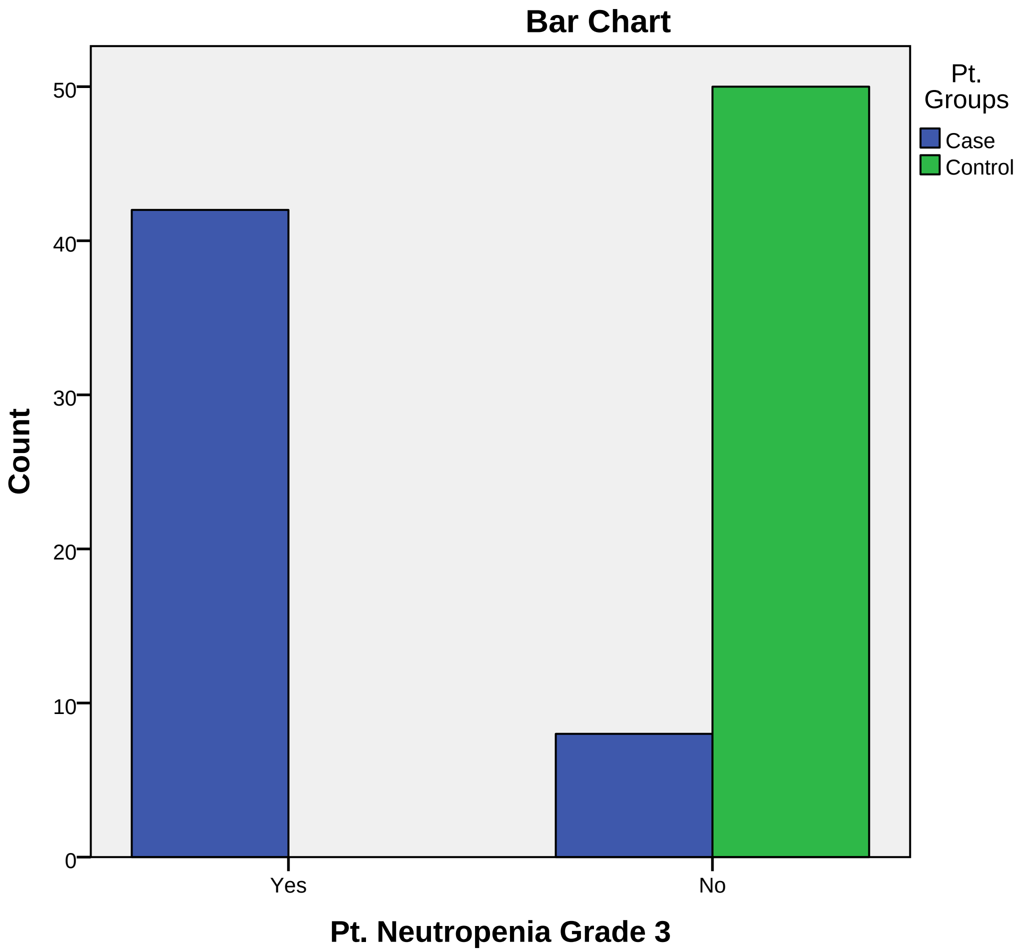

Pt. Neutropenia Grade 4 * Pt. Groups

Crosstab

Count

		Pt. Groups		
		Case	Control	Total
Pt. Neutropenia Grade 4	Yes	8	0	8
	No	42	50	92
Total		50	50	100

Chi-Square Tests

	Value	df	Asymptotic Significance (2-sided)	Exact Sig. (2-sided)	Exact Sig. (1-sided)
Pearson Chi-Square	8.696[a]	1	.003		
Continuity Correction[b]	6.658	1	.010		
Likelihood Ratio	11.787	1	.001		
Fisher's Exact Test				.006	.003
N of Valid Cases	100				

a. 2 cells (50.0%) have expected count less than 5. The minimum expected count is 4.00.

b. Computed only for a 2x2 table

Risk Estimate

	Value	95% Confidence Interval	
		Lower	Upper
For cohort Pt. Groups = Case	2.190	1.753	2.738
N of Valid Cases	100		

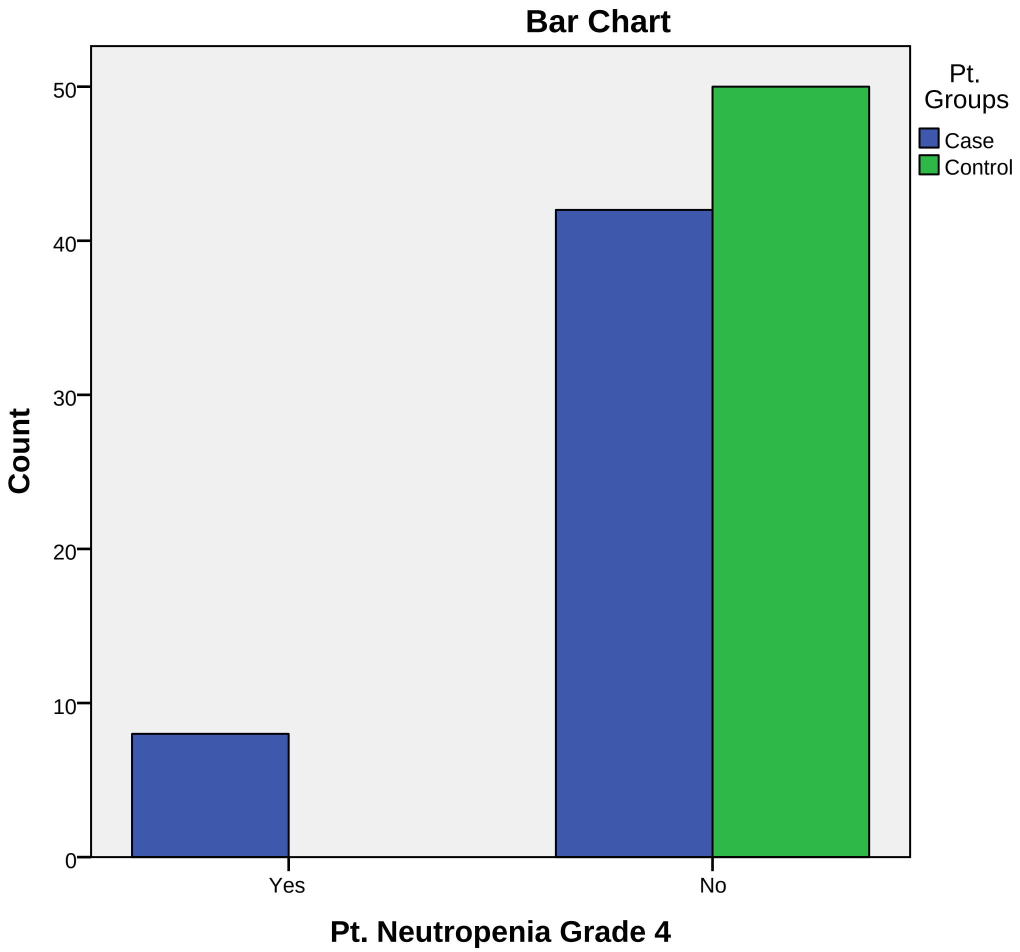

Estrogen Receptor * Pt. Groups

Crosstab

Count

		Pt. Groups		Total
		Case	Control	
Estrogen Receptor	ER+	36	38	74
	ER-	14	12	26
Total		50	50	100

Chi-Square Tests

	Value	df	Asymptotic Significance (2-sided)	Exact Sig. (2-sided)	Exact Sig. (1-sided)
Pearson Chi-Square	.208[a]	1	.648		
Continuity Correction[b]	.052	1	.820		
Likelihood Ratio	.208	1	.648		
Fisher's Exact Test				.820	.410
N of Valid Cases	100				

a. 0 cells (0.0%) have expected count less than 5. The minimum expected count is 13.00.

b. Computed only for a 2x2 table

Risk Estimate

	Value	95% Confidence Interval	
		Lower	Upper
Odds Ratio for Estrogen Receptor (ER+ / ER-)	.812	.332	1.989
For cohort Pt. Groups = Case	.903	.590	1.383
For cohort Pt. Groups = Control	1.113	.695	1.781
N of Valid Cases	100		

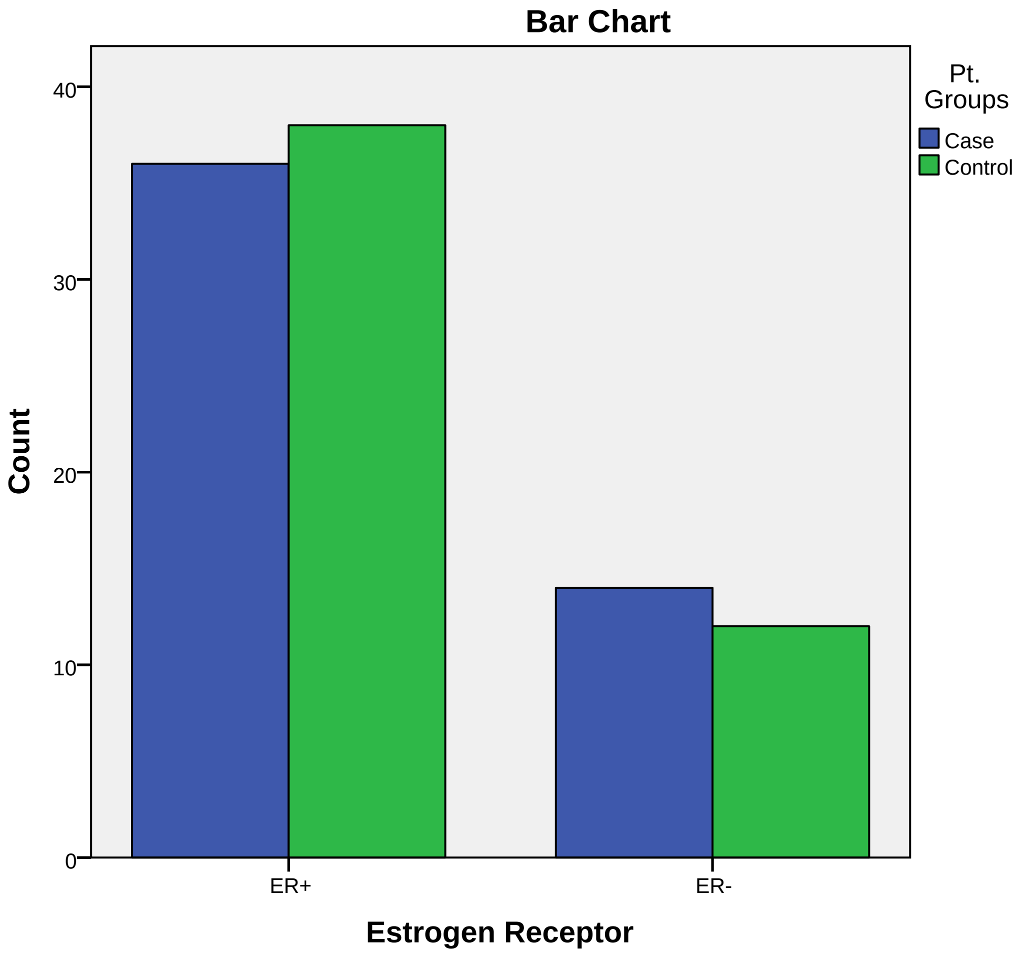

Progesterone Receptor * Pt. Groups

Crosstab

Count

		Pt. Groups		Total
		Case	Control	
Progesterone Receptor	PR+	33	37	70
	PR-	17	13	30
Total		50	50	100

Chi-Square Tests

	Value	df	Asymptotic Significance (2-sided)	Exact Sig. (2-sided)	Exact Sig. (1-sided)
Pearson Chi-Square	.762[a]	1	.383		
Continuity Correction[b]	.429	1	.513		
Likelihood Ratio	.764	1	.382		
Fisher's Exact Test				.513	.257
N of Valid Cases	100				

a. 0 cells (0.0%) have expected count less than 5. The minimum expected count is 15.00.

b. Computed only for a 2x2 table

Risk Estimate

	Value	95% Confidence Interval	
		Lower	Upper
Odds Ratio for Progesterone Receptor (PR+ / PR-)	.682	.288	1.614
For cohort Pt. Groups = Case	.832	.558	1.240
For cohort Pt. Groups = Control	1.220	.766	1.942
N of Valid Cases	100		

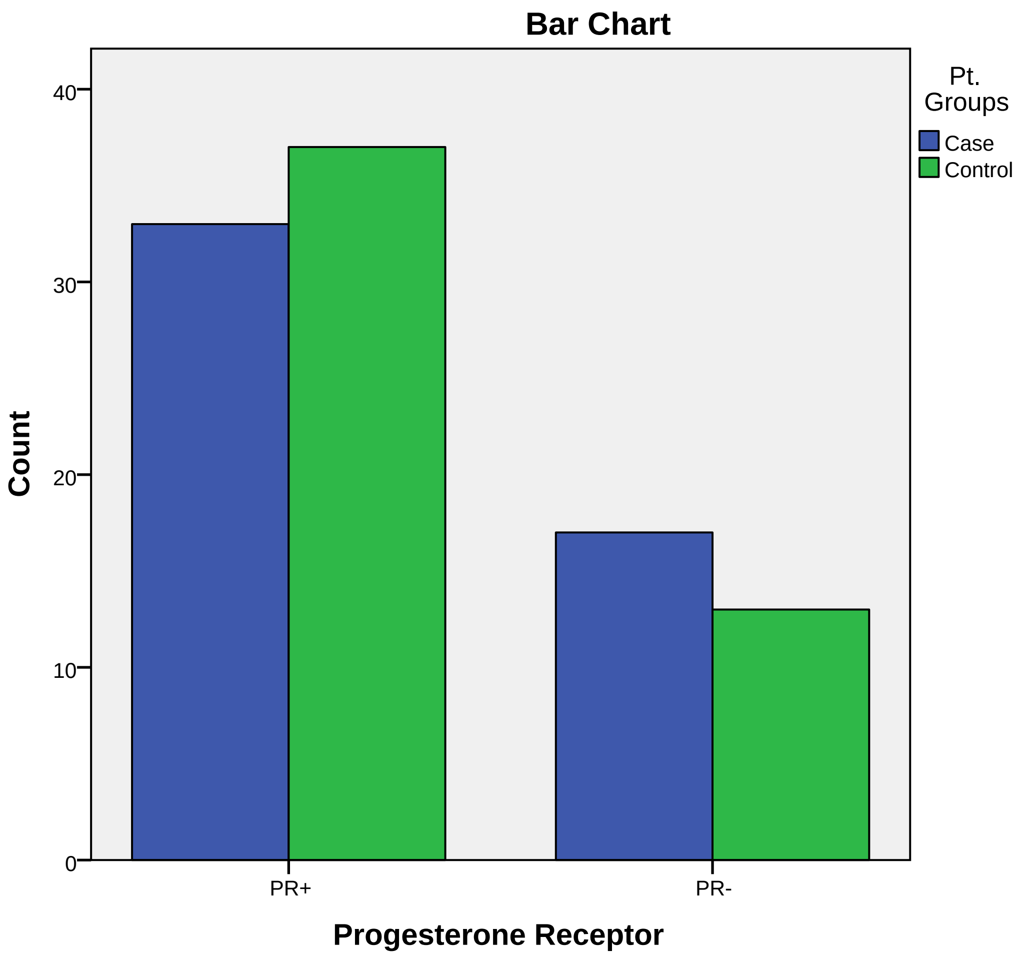

Human Epidermal Growth Factor Receptor 2 * Pt. Groups

Crosstab

Count

		Pt. Groups		Total
		Case	Control	
Human Epidermal Growth Factor Receptor 2	Her2+	25	27	52
	Her2-	25	23	48
Total		50	50	100

Chi-Square Tests

	Value	df	Asymptotic Significance (2-sided)	Exact Sig. (2-sided)	Exact Sig. (1-sided)
Pearson Chi-Square	.160[a]	1	.689		
Continuity Correction[b]	.040	1	.841		
Likelihood Ratio	.160	1	.689		
Fisher's Exact Test				.841	.421
N of Valid Cases	100				

a. 0 cells (0.0%) have expected count less than 5. The minimum expected count is 24.00.

b. Computed only for a 2x2 table

Risk Estimate

	Value	95% Confidence Interval	
		Lower	Upper
Odds Ratio for Human Epidermal Growth Factor Receptor 2 (Her2+ / Her2-)	.852	.388	1.868
For cohort Pt. Groups = Case	.923	.624	1.366
For cohort Pt. Groups = Control	1.084	.731	1.607
N of Valid Cases	100		

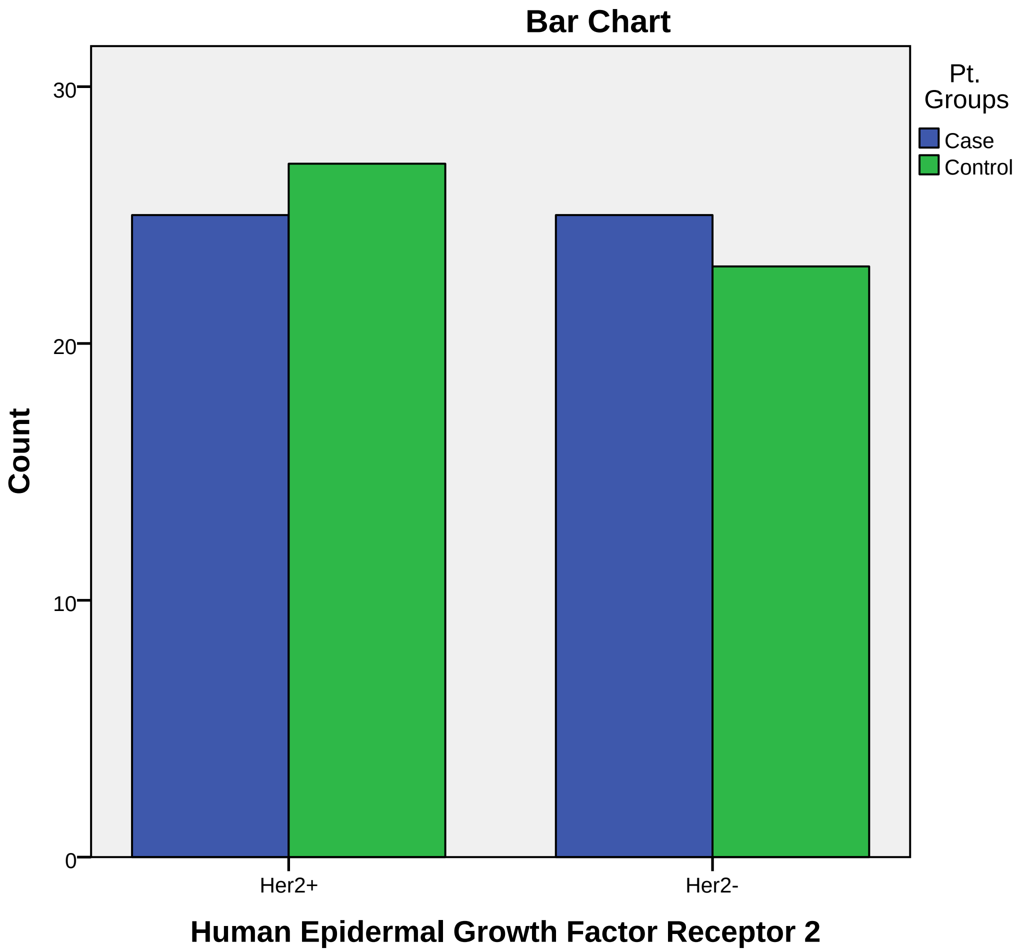

Chemotherapy Regimen * Pt. Groups

Crosstab

Count

		Pt. Groups		Total
		Case	Control	
Chemotherapy Regimen	ATC	31	33	64
	CAF	9	8	17
	TAC	10	9	19
Total		50	50	100

Chi-Square Tests

	Value	df	Asymptotic Significance (2-sided)
Pearson Chi-Square	.174[a]	2	.917
Likelihood Ratio	.174	2	.917
N of Valid Cases	100		

a. 0 cells (0.0%) have expected count less than 5. The minimum expected count is 8.50.

Risk Estimate

	Value
Odds Ratio for Chemotherapy Regimen (ATC / CAF)	[a]

a. Risk Estimate statistics cannot be computed. They are only computed for a 2*2 table without empty cells.

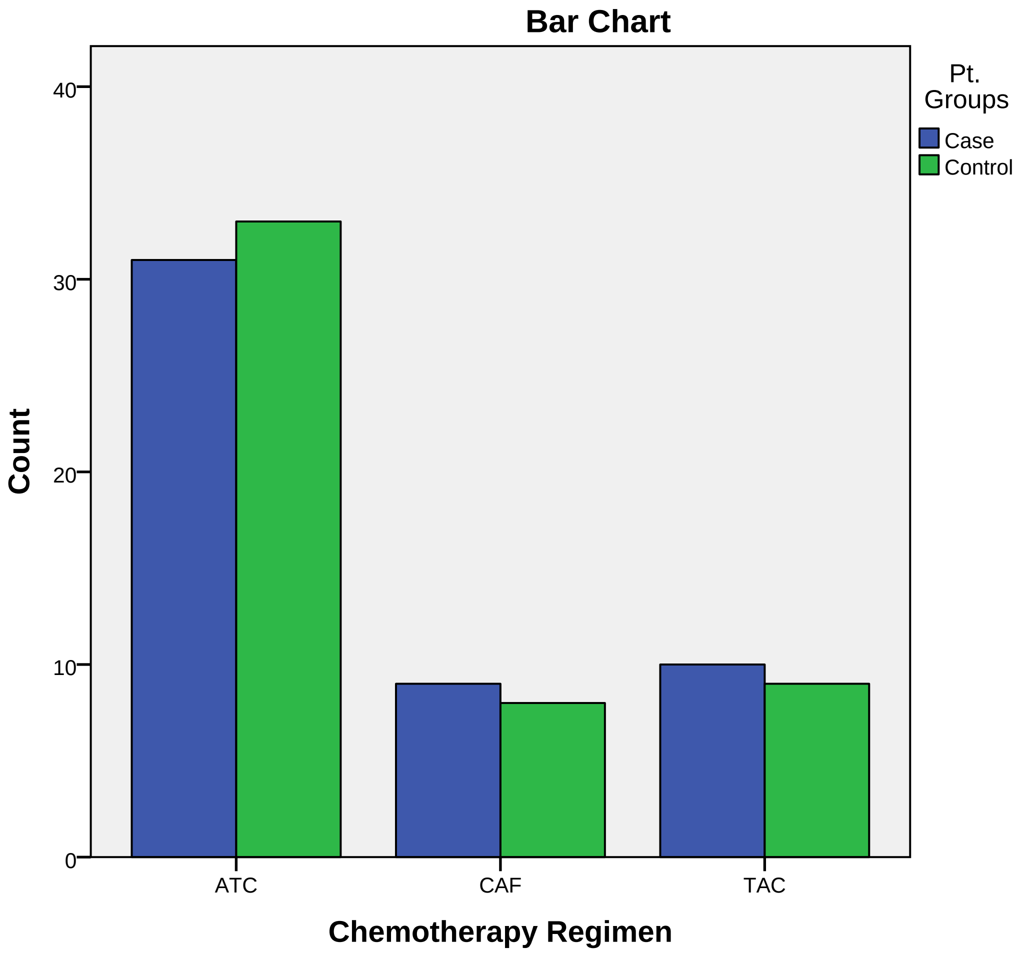

Other Disease * Pt. Groups

Crosstab

Count

		Pt. Groups		Total
		Case	Control	
Other Disease	Astma	2	1	3
	Arthritis	0	1	1
	Blood Hypertension	4	0	4
	Diabetes	2	6	8
	Heart	4	0	4
	Liver	4	0	4
	Normal	31	39	70
	Parkinson	0	1	1
	Tyroidism	3	2	5
Total		50	50	100

Chi-Square Tests

	Value	df	Asymptotic Significance (2-sided)
Pearson Chi-Square	17.448[a]	8	.026
Likelihood Ratio	22.959	8	.003
N of Valid Cases	100		

a. 16 cells (88.9%) have expected count less than 5. The minimum expected count is .50.

Risk Estimate

	Value
Odds Ratio for Other Disease (Astma / Arthritis)	[a]

a. Risk Estimate statistics cannot be computed. They are only computed for a 2*2 table without empty cells.

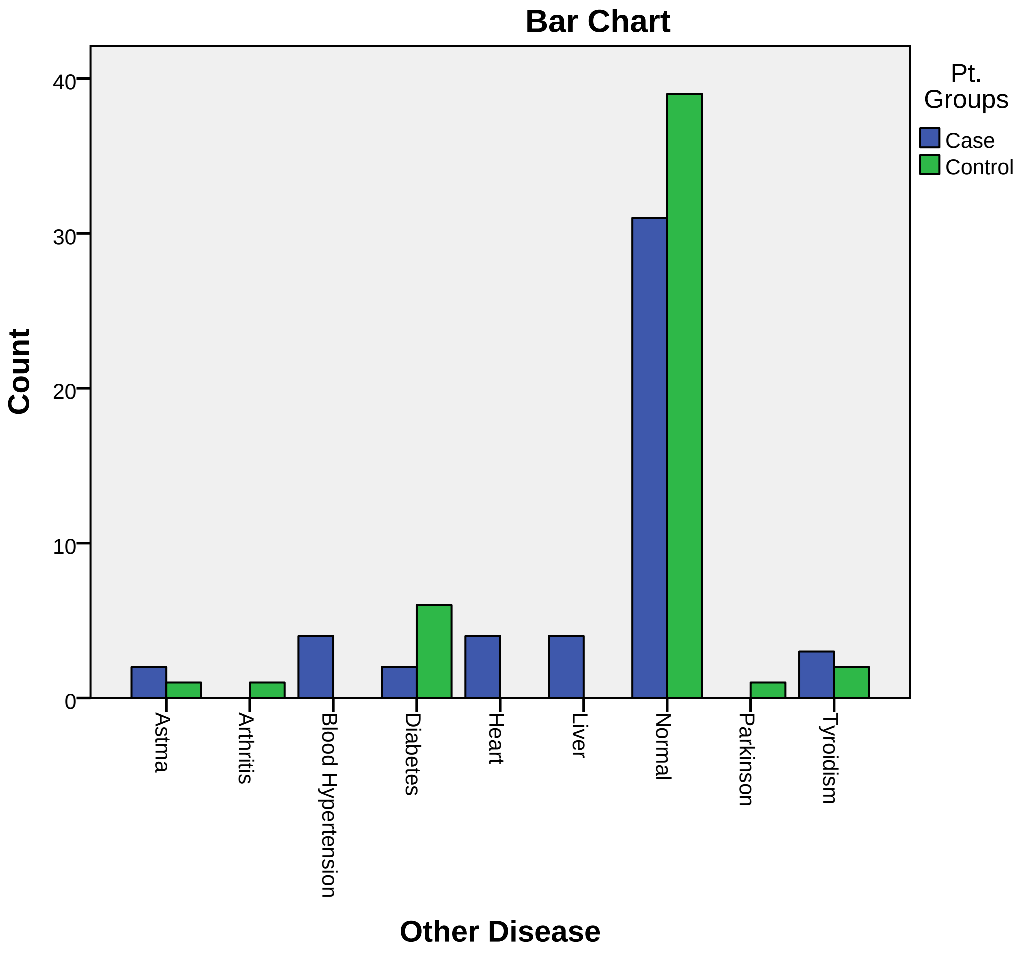

Ductal Carcinoma * Pt. Groups

Crosstab

Count

		Pt. Groups		Total
		Case	Control	
Ductal Carcinoma	Yes	48	48	96
	No	2	2	4
Total		50	50	100

Chi-Square Tests

	Value	df	Asymptotic Significance (2-sided)	Exact Sig. (2-sided)	Exact Sig. (1-sided)
Pearson Chi-Square	.000[a]	1	1.000		
Continuity Correction[b]	.000	1	1.000		
Likelihood Ratio	.000	1	1.000		
Fisher's Exact Test				1.000	.691
N of Valid Cases	100				

a. 2 cells (50.0%) have expected count less than 5. The minimum expected count is 2.00.

b. Computed only for a 2x2 table

Risk Estimate

	Value	95% Confidence Interval	
		Lower	Upper
Odds Ratio for Ductal Carcinoma (Yes / No)	1.000	.135	7.392
For cohort Pt. Groups = Case	1.000	.368	2.719
For cohort Pt. Groups = Control	1.000	.368	2.719
N of Valid Cases	100		

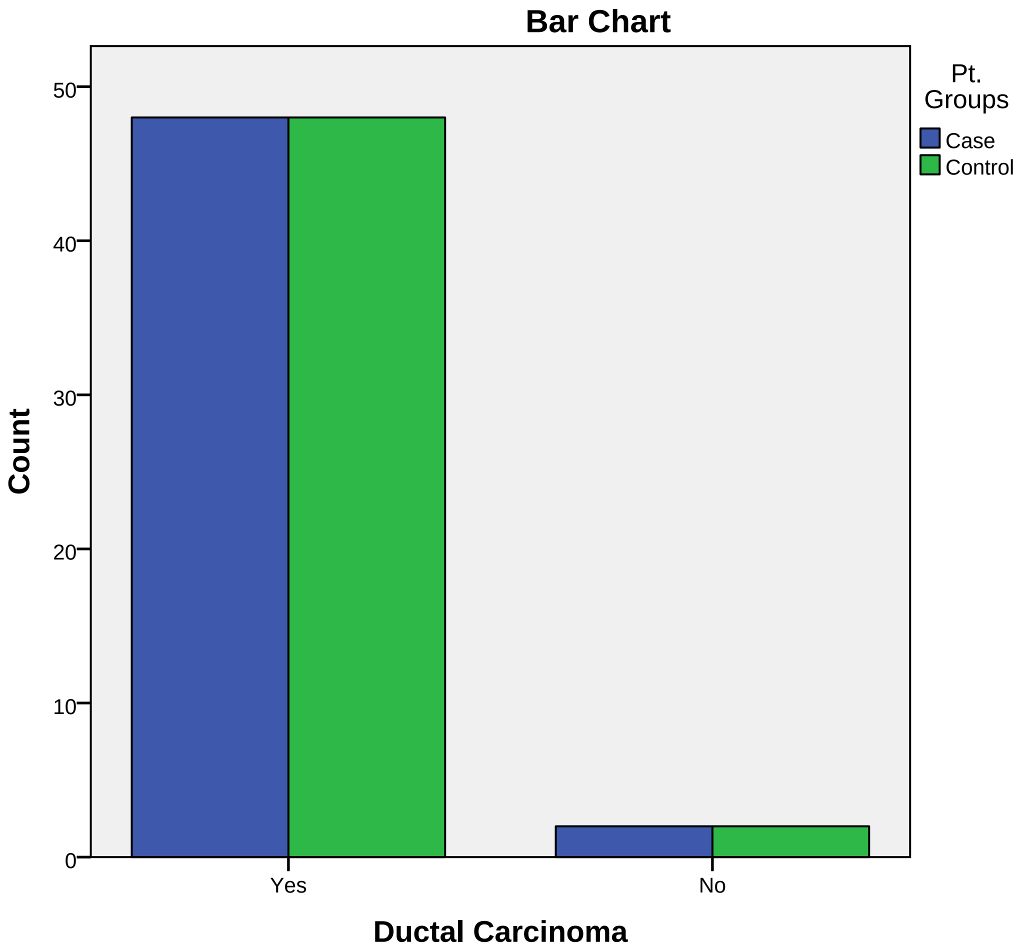

Lobular Carcinoma * Pt. Groups

Crosstab

Count

		Pt. Groups		Total
		Case	Control	
Lobular Carcinoma	Yes	2	2	4
	No	48	48	96
Total		50	50	100

Chi-Square Tests

	Value	df	Asymptotic Significance (2-sided)	Exact Sig. (2-sided)	Exact Sig. (1-sided)
Pearson Chi-Square	.000[a]	1	1.000		
Continuity Correction[b]	.000	1	1.000		
Likelihood Ratio	.000	1	1.000		
Fisher's Exact Test				1.000	.691
N of Valid Cases	100				

a. 2 cells (50.0%) have expected count less than 5. The minimum expected count is 2.00.

b. Computed only for a 2x2 table

Risk Estimate

	Value	95% Confidence Interval	
		Lower	Upper
Odds Ratio for Lobular Carcinoma (Yes / No)	1.000	.135	7.392
For cohort Pt. Groups = Case	1.000	.368	2.719
For cohort Pt. Groups = Control	1.000	.368	2.719
N of Valid Cases	100		

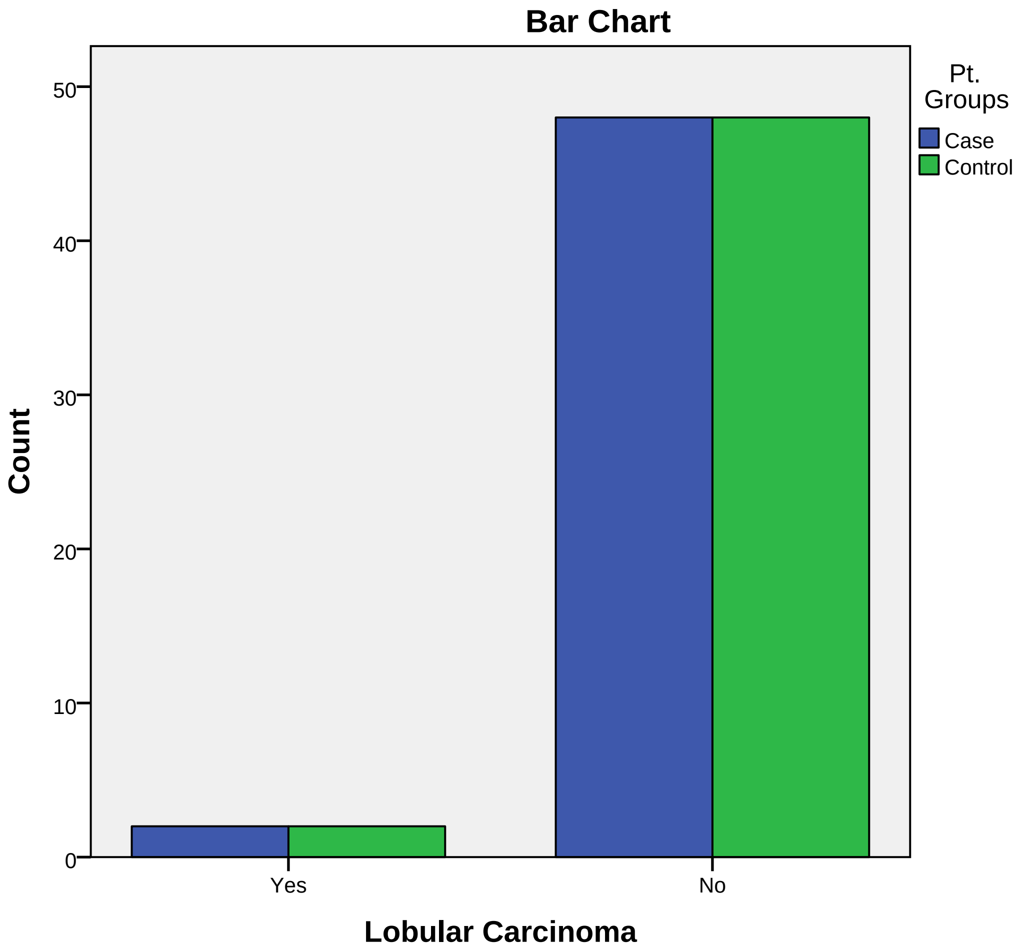

Cancer Stage IA * Pt. Groups

Crosstab

Count

		Pt. Groups		Total
		Case	Control	
Cancer Stage IA	Yes	6	5	11
	No	44	45	89
Total		50	50	100

Chi-Square Tests

	Value	df	Asymptotic Significance (2-sided)	Exact Sig. (2-sided)	Exact Sig. (1-sided)
Pearson Chi-Square	.102[a]	1	.749		
Continuity Correction[b]	.000	1	1.000		
Likelihood Ratio	.102	1	.749		
Fisher's Exact Test				1.000	.500
N of Valid Cases	100				

a. 0 cells (0.0%) have expected count less than 5. The minimum expected count is 5.50.

b. Computed only for a 2x2 table

Risk Estimate

	Value	95% Confidence Interval	
		Lower	Upper
Odds Ratio for Cancer Stage IA (Yes / No)	1.227	.349	4.316
For cohort Pt. Groups = Case	1.103	.618	1.968
For cohort Pt. Groups = Control	.899	.456	1.773
N of Valid Cases	100		

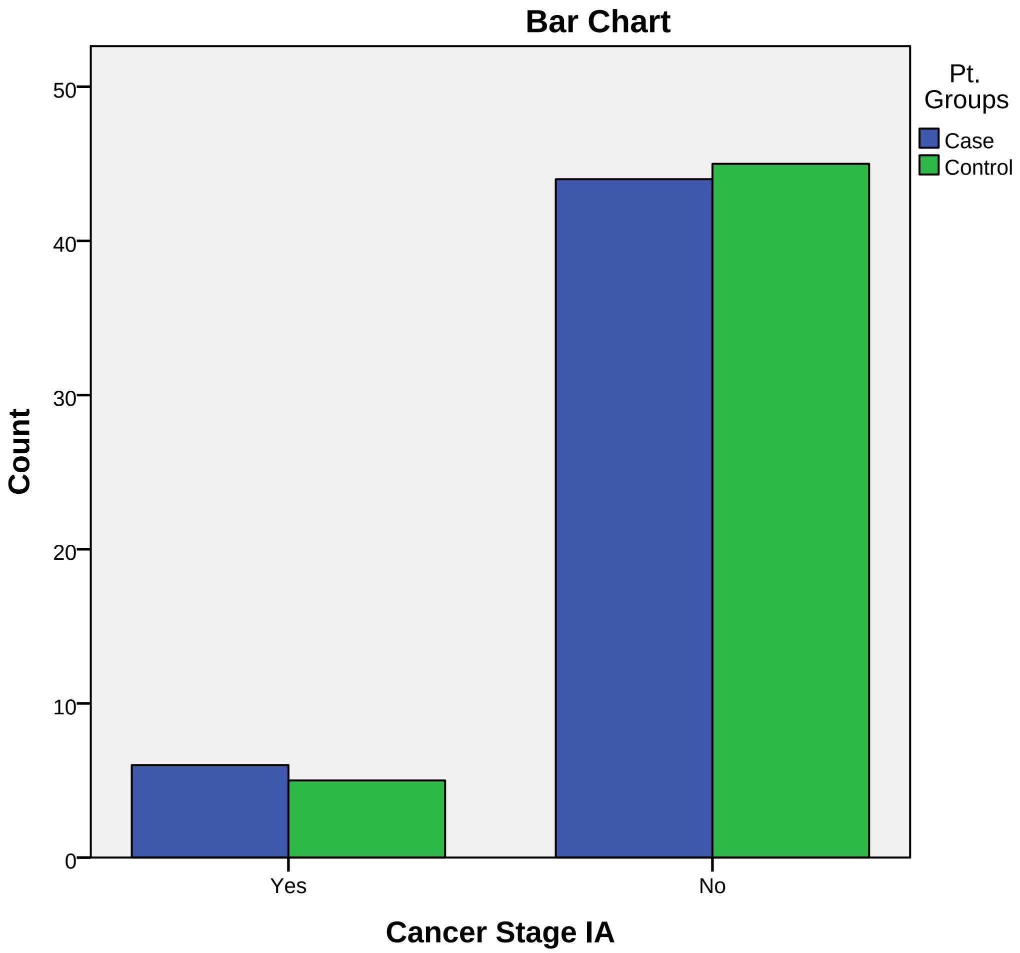

Cancer Stage IIA * Pt. Groups

Crosstab

Count

		Pt. Groups		Total
		Case	Control	
Cancer Stage IIA	Yes	9	19	28
	No	41	31	72
Total		50	50	100

Chi-Square Tests

	Value	df	Asymptotic Significance (2-sided)	Exact Sig. (2-sided)	Exact Sig. (1-sided)
Pearson Chi-Square	4.960[a]	1	.026		
Continuity Correction[b]	4.018	1	.045		
Likelihood Ratio	5.045	1	.025		
Fisher's Exact Test				.044	.022
N of Valid Cases	100				

a. 0 cells (0.0%) have expected count less than 5. The minimum expected count is 14.00.

b. Computed only for a 2x2 table

Risk Estimate

	Value	95% Confidence Interval	
		Lower	Upper
Odds Ratio for Cancer Stage IIA (Yes / No)	.358	.143	.899
For cohort Pt. Groups = Case	.564	.318	1.003
For cohort Pt. Groups = Control	1.576	1.091	2.278
N of Valid Cases	100		

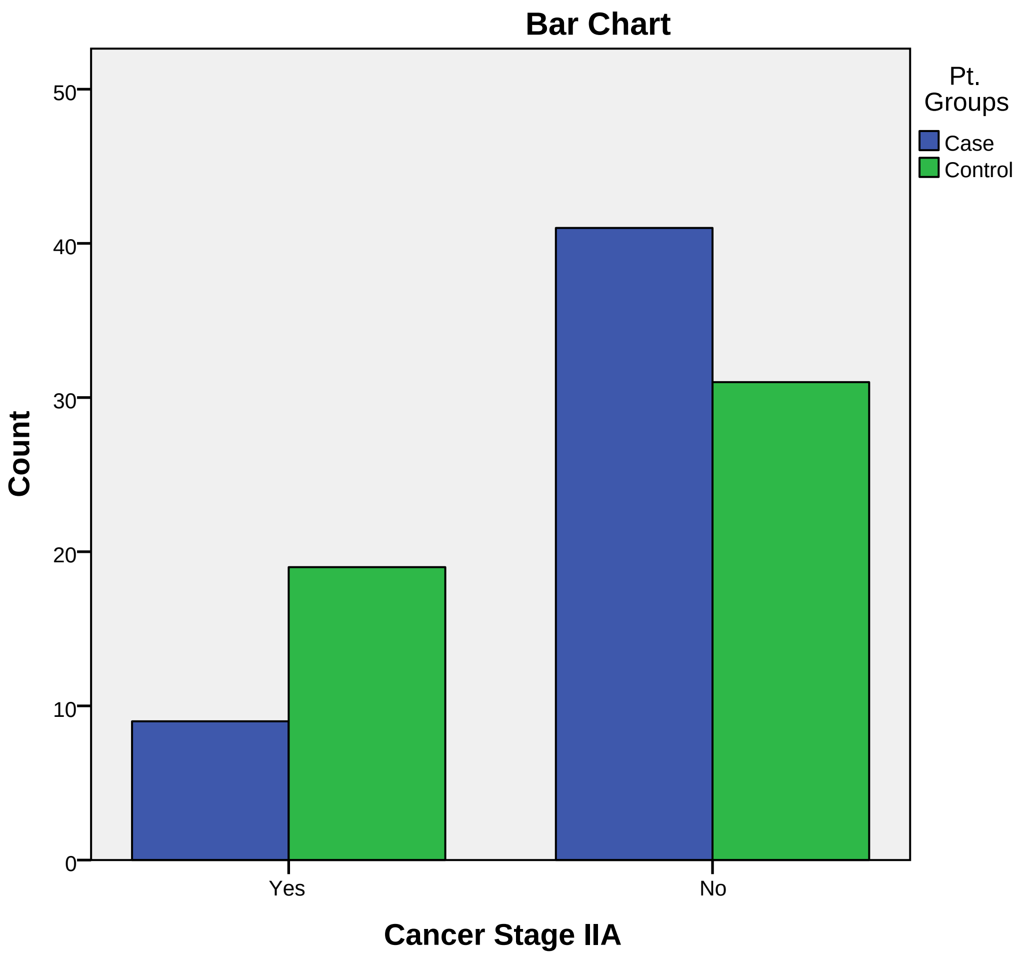

Cancer Stage IIB * Pt. Groups

Crosstab

Count

		Pt. Groups		Total
		Case	Control	
Cancer Stage IIB	Yes	10	13	23
	No	40	37	77
Total		50	50	100

Chi-Square Tests

	Value	df	Asymptotic Significance (2-sided)	Exact Sig. (2-sided)	Exact Sig. (1-sided)
Pearson Chi-Square	.508[a]	1	.476		
Continuity Correction[b]	.226	1	.635		
Likelihood Ratio	.509	1	.475		
Fisher's Exact Test				.635	.318
N of Valid Cases	100				

a. 0 cells (0.0%) have expected count less than 5. The minimum expected count is 11.50.

b. Computed only for a 2x2 table

Risk Estimate

	Value	95% Confidence Interval	
		Lower	Upper
Odds Ratio for Cancer Stage IIB (Yes / No)	.712	.279	1.818
For cohort Pt. Groups = Case	.837	.501	1.398
For cohort Pt. Groups = Control	1.176	.767	1.803
N of Valid Cases	100		

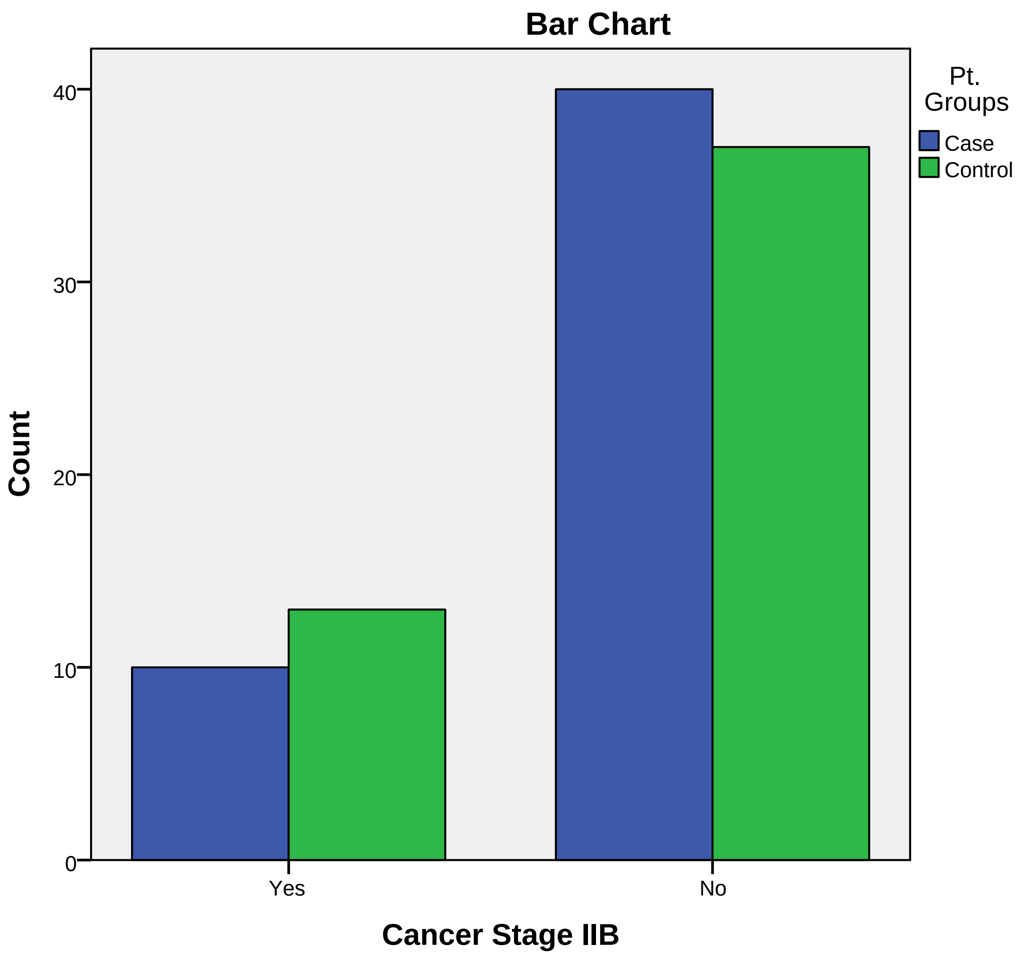

Cancer Stage IIIA * Pt. Groups

Crosstab

Count

		Pt. Groups		Total
		Case	Control	
Cancer Stage IIIA	Yes	15	10	25
	No	35	40	75
Total		50	50	100

Chi-Square Tests

	Value	df	Asymptotic Significance (2-sided)	Exact Sig. (2-sided)	Exact Sig. (1-sided)
Pearson Chi-Square	1.333[a]	1	.248		
Continuity Correction[b]	.853	1	.356		
Likelihood Ratio	1.340	1	.247		
Fisher's Exact Test				.356	.178
N of Valid Cases	100				

a. 0 cells (0.0%) have expected count less than 5. The minimum expected count is 12.50.

b. Computed only for a 2x2 table

Risk Estimate

	Value	95% Confidence Interval	
		Lower	Upper
Odds Ratio for Cancer Stage IIIA (Yes / No)	1.714	.683	4.301
For cohort Pt. Groups = Case	1.286	.861	1.920
For cohort Pt. Groups = Control	.750	.444	1.267
N of Valid Cases	100		

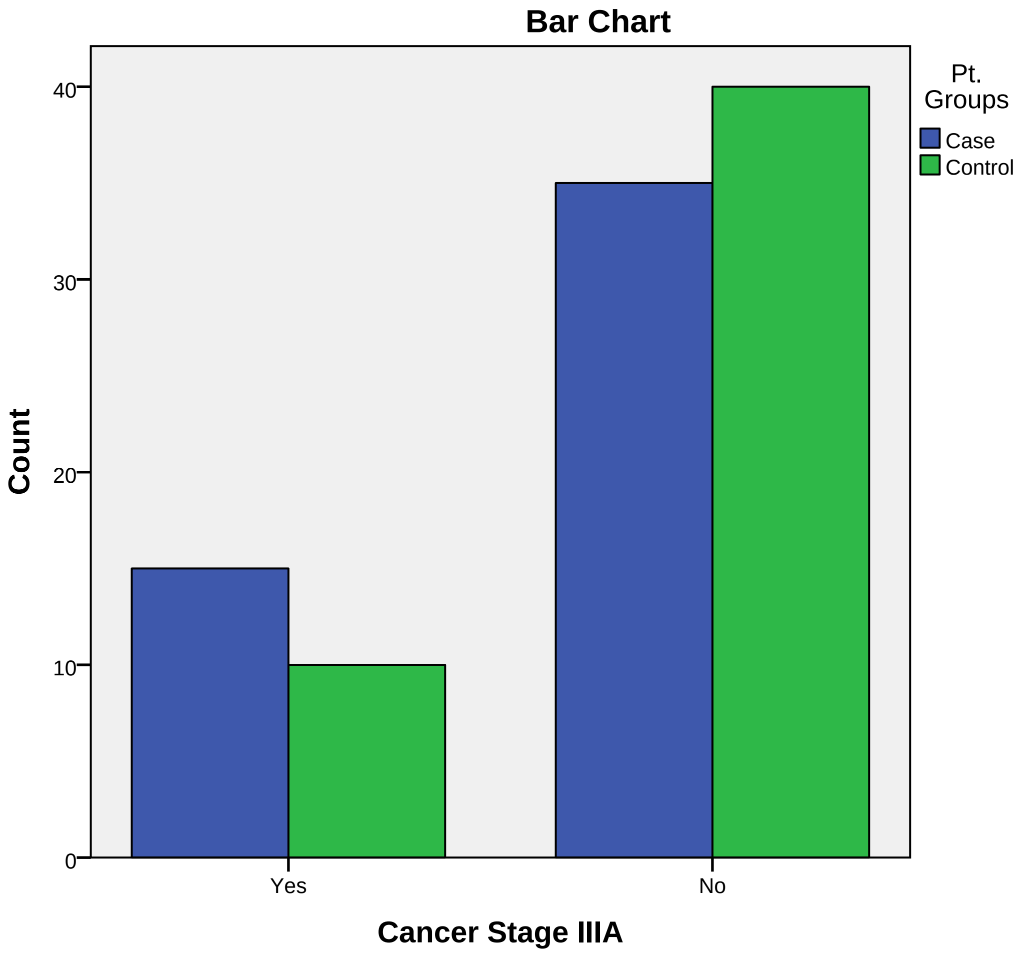

Cancer Stage IIIC * Pt. Groups

Crosstab

Count

		Pt. Groups		Total
		Case	Control	
Cancer Stage IIIC	Yes	10	3	13
	No	40	47	87
Total		50	50	100

Chi-Square Tests

	Value	df	Asymptotic Significance (2-sided)	Exact Sig. (2-sided)	Exact Sig. (1-sided)
Pearson Chi-Square	4.332[a]	1	.037		
Continuity Correction[b]	3.183	1	.074		
Likelihood Ratio	4.540	1	.033		
Fisher's Exact Test				.071	.036
N of Valid Cases	100				

a. 0 cells (0.0%) have expected count less than 5. The minimum expected count is 6.50.

b. Computed only for a 2x2 table

Risk Estimate

	Value	95% Confidence Interval	
		Lower	Upper
Odds Ratio for Cancer Stage IIIC (Yes / No)	3.917	1.008	15.220
For cohort Pt. Groups = Case	1.673	1.150	2.434
For cohort Pt. Groups = Control	.427	.155	1.174
N of Valid Cases	100		

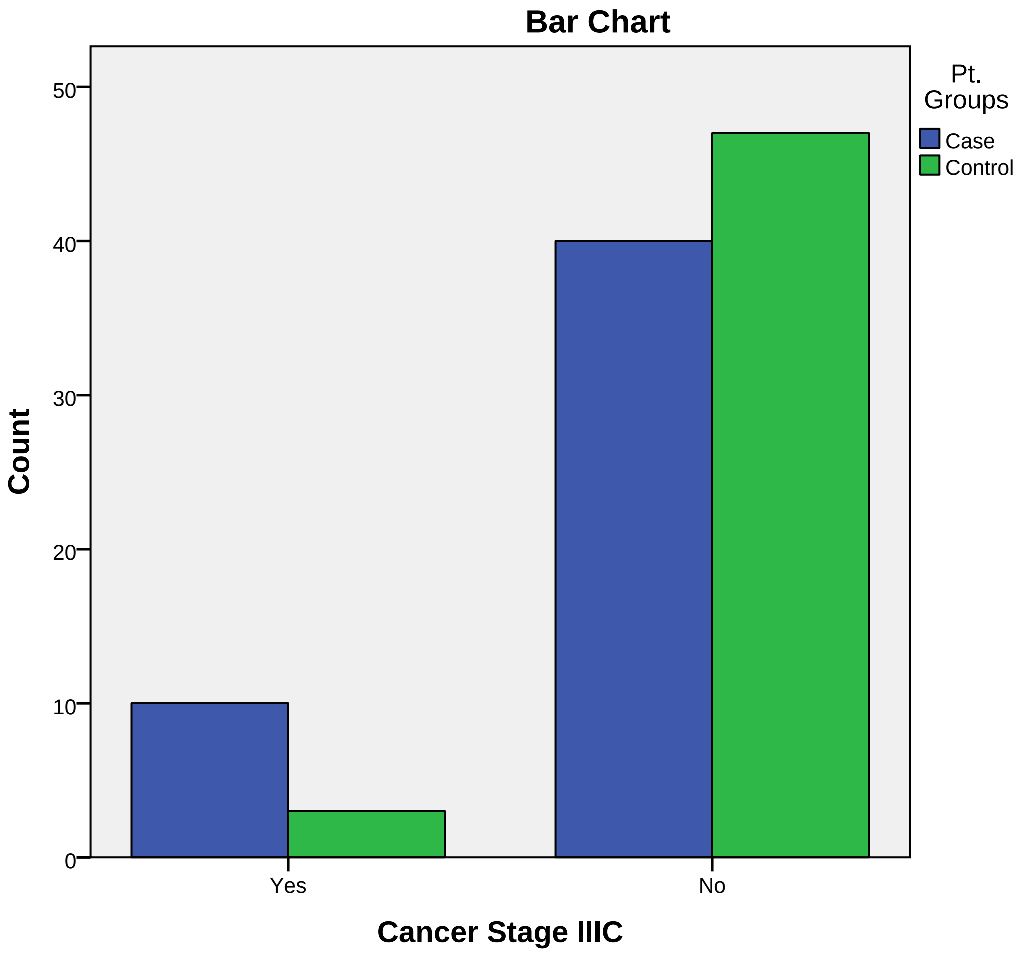

Tumor Grade I * Pt. Groups

Crosstab

Count

		Pt. Groups		Total
		Case	Control	
Tumor Grade I	Yes	5	4	9
	No	45	46	91
Total		50	50	100

Chi-Square Tests

	Value	df	Asymptotic Significance (2-sided)	Exact Sig. (2-sided)	Exact Sig. (1-sided)
Pearson Chi-Square	.122[a]	1	.727		
Continuity Correction[b]	.000	1	1.000		
Likelihood Ratio	.122	1	.727		
Fisher's Exact Test				1.000	.500
N of Valid Cases	100				

a. 2 cells (50.0%) have expected count less than 5. The minimum expected count is 4.50.

b. Computed only for a 2x2 table

Risk Estimate

	Value	95% Confidence Interval	
		Lower	Upper
Odds Ratio for Tumor Grade I (Yes / No)	1.278	.322	5.066
For cohort Pt. Groups = Case	1.123	.604	2.089
For cohort Pt. Groups = Control	.879	.412	1.877
N of Valid Cases	100		

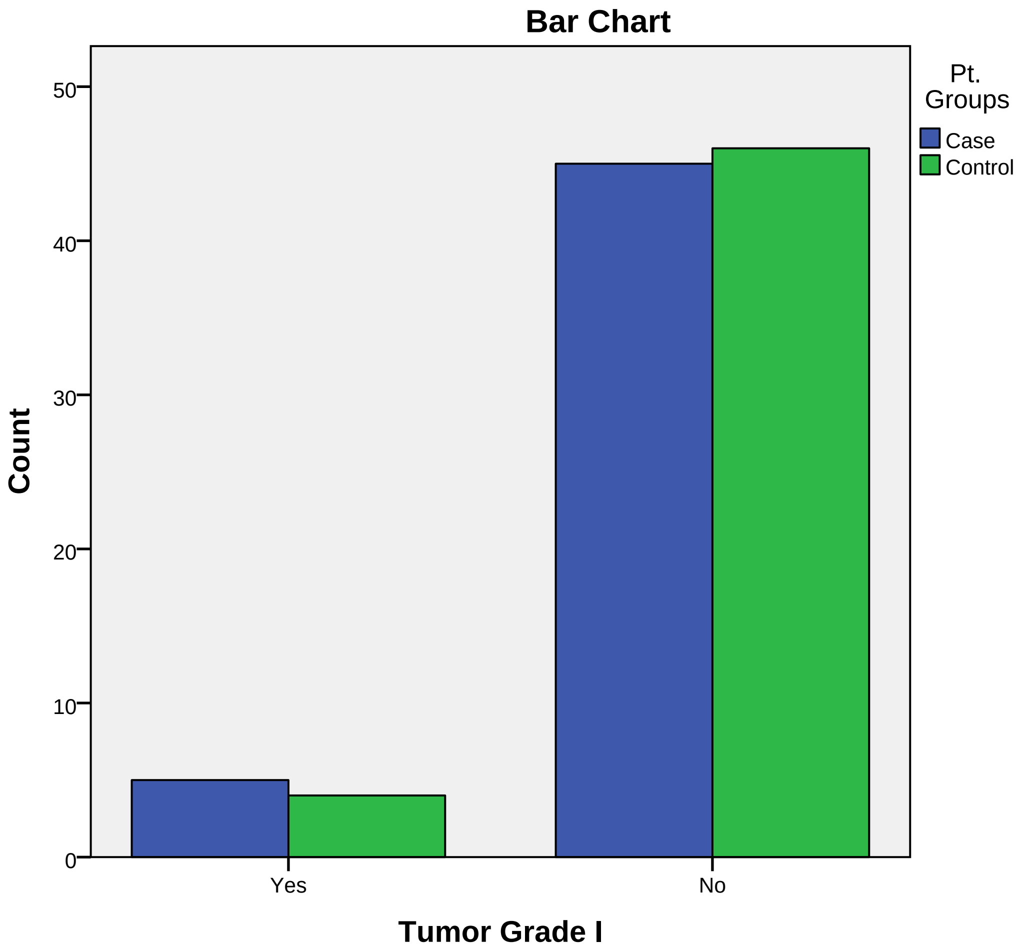

Tumor Grade II * Pt. Groups

Crosstab

Count

		Pt. Groups		Total
		Case	Control	
Tumor Grade II	Yes	36	36	72
	No	14	14	28
Total		50	50	100

Chi-Square Tests

	Value	df	Asymptotic Significance (2-sided)	Exact Sig. (2-sided)	Exact Sig. (1-sided)
Pearson Chi-Square	.000[a]	1	1.000		
Continuity Correction[b]	.000	1	1.000		
Likelihood Ratio	.000	1	1.000		
Fisher's Exact Test				1.000	.588
N of Valid Cases	100				

a. 0 cells (0.0%) have expected count less than 5. The minimum expected count is 14.00.

b. Computed only for a 2x2 table

Risk Estimate

	Value	95% Confidence Interval	
		Lower	Upper
Odds Ratio for Tumor Grade II (Yes / No)	1.000	.418	2.394
For cohort Pt. Groups = Case	1.000	.646	1.547
For cohort Pt. Groups = Control	1.000	.646	1.547
N of Valid Cases	100		

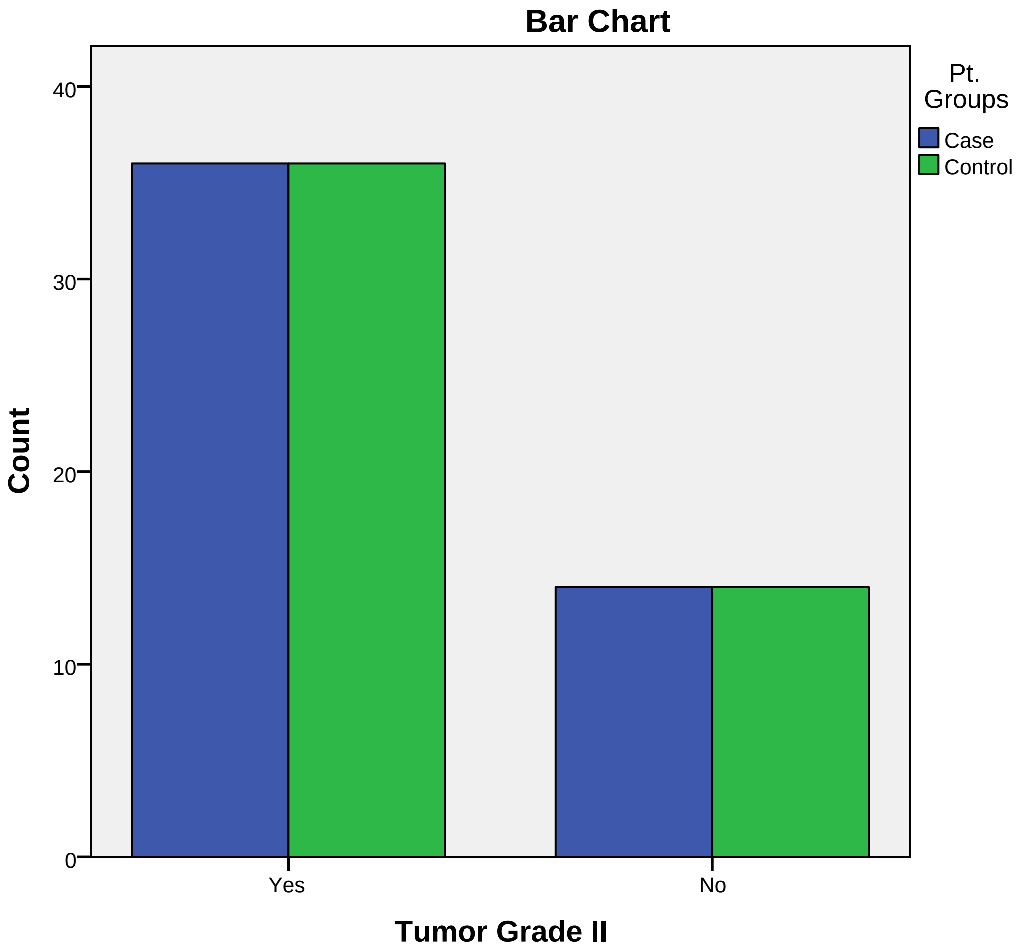

Tumor Grade III * Pt. Groups

Crosstab

Count

		Pt. Groups		Total
		Case	Control	
Tumor Grade III	Yes	9	10	19
	No	41	40	81
Total		50	50	100

Chi-Square Tests

	Value	df	Asymptotic Significance (2-sided)	Exact Sig. (2-sided)	Exact Sig. (1-sided)
Pearson Chi-Square	.065[a]	1	.799		
Continuity Correction[b]	.000	1	1.000		
Likelihood Ratio	.065	1	.799		
Fisher's Exact Test				1.000	.500
N of Valid Cases	100				

a. 0 cells (0.0%) have expected count less than 5. The minimum expected count is 9.50.

b. Computed only for a 2x2 table

Risk Estimate

	Value	95% Confidence Interval	
		Lower	Upper
Odds Ratio for Tumor Grade III (Yes / No)	.878	.323	2.388
For cohort Pt. Groups = Case	.936	.556	1.575
For cohort Pt. Groups = Control	1.066	.659	1.723
N of Valid Cases	100		

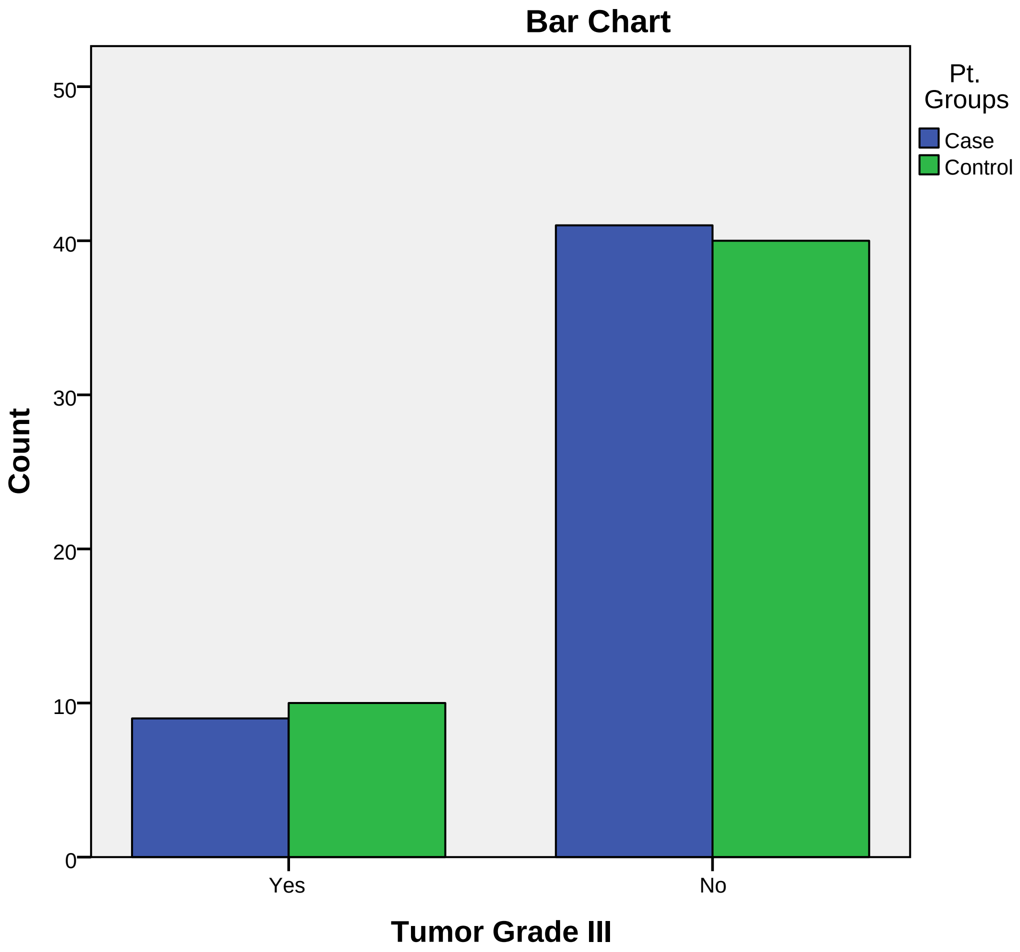

Bar Chart
Pt.
Groups
Case
Control
50
40
30
20
10
0
Count
Yes
No
Tumor Grade III
